# FeCl_3_∙6H_2_O/TMSBr-Catalyzed Rapid Synthesis of Dihydropyrimidinones and Dihydropyrimidinethiones under Microwave Irradiation

**DOI:** 10.3390/molecules22091503

**Published:** 2017-09-11

**Authors:** Fei Zhao, Xiuwen Jia, Pinyi Li, Jingwei Zhao, Jun Huang, Honglian Li, Lin Li

**Affiliations:** Antibiotics Research and Re-Evaluation Key Laboratory of Sichuan Province, Sichuan Industrial Institute of Antibiotics, Chengdu University, 168 Hua Guan Road, Chengdu 610052, China; echo19930319@163.com (X.J.); 18349336094@163.com (P.L.); huizi0268616@sina.com.cn (J.Z.); 13183854809@163.com (J.H.); 13281813368@163.com (H.L.); ll18328653533@126.com (L.L.)

**Keywords:** FeCl_3_∙6H_2_O, TMSBr, dihydropyrimidinones, dihydropyrimidinethiones, microwave irradiation

## Abstract

An efficient and practical protocol has been developed to synthesize dihydropyrimidinones and dihydropyrimidinethiones through FeCl_3_∙6H_2_O/TMSBr-catalyzed three-component cyclocondensation under microwave irradiation. This approach features high yields, broad substrate scope, short reaction time, mild reaction conditions, operational simplicity and easy work-up, thus affording a versatile method for the synthesis of dihydropyrimidinones and dihydropyrimidinethiones.

## 1. Introduction

Dihydropyrimidinones and dihydropyrimidinethiones have received great attention in synthetic organic chemistry because of their ubiquitous presence in a large number of natural products and pharmaceutical agents [1,2,3,4,5,6,7,8,9,10]. For example, they act as key components in natural marine alkaloids such as batzelladine A-I [11,12,13], ptilocaulin [14], and saxitoxin [15]. Moreover, they exhibit a broad spectrum of pharmacological activities such as antibacterial [16], antitumor [17,18,19,20], anti-inflammatory [20], antiviral [21], and antihypertensive activities [22,23]. They are also known as calcium channel blockers [24,25,26,27] and α_1A_-adrenergic receptor (α_1A_-AR) antagonists [28,29]. In addition, dihydropyrimidinones display as a key precursor in the synthesis of pyrimidine bases which constitute the basic skeleton of nucleic acids [30]. Therefore, an efficient access to these two structures is highly desirable for both organic synthesis and drug discovery.

The first synthetic method for the preparation of dihydropyrimidinones and dihydropyrimidinethiones was reported by Biginelli in 1893 [31]. However, this method suffered from low yields and the usage of strong acids. Consequently, improved procedures, including the employment of Lewis acid catalysts [32,33,34,35,36,37,38,39,40,41,42,43,44,45,46,47,48,49,50,51,52,53,54,55,56], bases [57,58], ionic liquids [59,60,61,62], ultrasound irradiation [63], and nanocomposites [64,65,66] have been developed. Despite the remarkable achievements made, however, many of these methods still suffer from major or minor drawbacks, such as long reaction time, harsh reaction conditions, low yields, the stoichiometric requirements of the metal catalysts and the involvement of expensive or toxic reagents. Therefore, the development of a faster, milder, high-yielding and environmentally benign approach for the synthesis of dihydropyrimidinones and dihydropyrimidinethiones is still of great significance. Herein, we present our efforts towards FeCl_3_∙6H_2_O/TMSBr- (TMSBr = Bromotrimethylsilane) catalyzed three-component cyclocondensation under microwave irradiation to synthesize dihydropyrimidinones and dihydropyrimidinethiones. Our protocol features high yields, broad substrate scope, short reaction time, mild reaction conditions, operational simplicity and easy work-up, thus affording a rapid and convenient approach for the synthesis of dihydropyrimidinones and dihydropyrimidinethiones.

## 2. Results and Discussion

We began our study by investigating the reaction of 1-tetralone (**1a**), benzaldehyde (**2a**) and thiourea (**3a**) in CH_3_CN at 90 °C in an oil bath for 10 h employing FeCl_3_∙6H_2_O as the catalyst, considering the potential of FeCl_3_∙6H_2_O in Biginelli-like reactions [45,67,68] (Table 1, entry 1). Pleasingly, the desired product **4a** was obtained, albeit with low yield. Considering that microwave-assisted organic synthesis (MAOS) is time and energy-saving [69,70,71] and its applications in Biginelli-like reactions [57,72,73,74,75,76,77,78,79,80,81], we then chose this technology to conduct the three-component condensation reaction. As a result, a similar yield was obtained under the same catalytic conditions when the reaction was carried out under microwave irradiation for just 2 h (Table 1, entry 2). Then, we tried to optimize the reaction conditions under microwave heating. At first, various catalysts such as ZnCl_2_, FeSO_4_∙7H_2_O, CuBr_2_, and AlCl_3_ were evaluated (Table 1, entries 3–6), and none of them exhibited a higher catalytic performance than FeCl_3_∙6H_2_O. Next, employing FeCl_3_∙6H_2_O as the catalyst, a series of additives were added and screened in order to improve the reaction yield (Table 1, entries 7–12). Both BF_3_∙OEt_2_ and BBr_3_ caused no obvious enhancement of the reaction yield (Table 1, entries 7 and 8). As TMS-X-type (X = Cl, I, OTf) compounds were proved to be efficient reagents which could significantly promote Biginelli-like reactions [44,45,56,82,83,84,85,86,87,88,89,90,91,92,93,94,95,96,97,98,99,100,101,102], we then explored the effects of this kind of additive. We found that TMSOTf, TMSCl, TMSBr and TMSI could improve the yield to different degrees (Table 1, entries 9–12). To our delight, TMSBr was found to be the most efficient additive, with which product **4a** was obtained in 88% yield by filtration (Table 1, entry 11). This might be because TMSBr could activate the carbonyl group of **1a** to promote the reaction [103,104,105]. Subsequently, a further screening of the solvents revealed that increasing the polarity of the solvent generally had a positive effect on the reaction yield (Table 1, entry 13–16), and CH_3_CN displayed as the best choice to promote the transformation, although ethanol could be an alternative solvent with which a slight lower yield (84%) was observed (Table 1, entry 16). In addition, solvent-free conditions were also tested, and only a moderate yield (52%) was obtained because of the recovery of the materials (Table 1, entry 17). By contrast, a lower yield (80%) was observed when the reaction was heated with an oil bath for 8 h under the optimal reaction conditions because of the incomplete consumption of the substrates (Table 1, entry 18), and it took 10 hours to finish the reaction to obtain a comparable yield (87%) using an oil bath (Table 1, entry 19). These results highlighted the efficiency of microwave irradiation. In this way, FeCl_3_∙6H_2_O/TMSBr-catalyzed synthesis of dihydropyrimidinethiones through three-component cyclocondensation under microwave heating was developed.

After determining the optimal reaction conditions, we then examined the general applicability of this process. In general, various substituted 3,4-dihydropyrimidin-2(1*H*)-thiones were easily prepared in good to high yields by the reaction of ketones, benzaldehydes and thiourea under the catalysis of FeCl_3_∙6H_2_O/TMSBr (Table 2). The reactions of benzaldehydes carrying electron-donating groups (Me, MeO) furnished the corresponding products **4b**–**4d** in 80%–86% yields (Table 2, entries 2–4). The protocol was also compatible with benzaldehydes bearing electron-withdrawing groups (CN, COOMe) and afforded the desired products **4e**–**4f** in good to high yields (Table 2, entries 5 and 6). Halogens (F, Cl, Br) were tolerated well and excellent yields (89%–90%) were obtained (Table 2, entries 7–9). Subsequently, the substituents on the 1-tetralones were investigated. As a result, the reactions of 1-tetralones with electron-donating group (MeO), halogen (Br) and electron-withdrawing group (NO_2_) on the benzene ring gave the corresponding products **4j**–**4l** in high yields (Table 2, entries 10–12). In addition, a high yield (89%) was observed when 1-indanone was subjected to the optimal reaction conditions (Table 2, entries 13).

Next, a wide range of structurally diverse ketones, benzaldehydes and urea were subjected to the optimal reaction conditions to produce the corresponding 3,4-dihydropyrimidin-2(1*H*)-ones in high yields (Table 3). The reaction of benzaldehyde furnished the product **6a** in 90% yield (Table 3, entry 1). Benzaldehydes with electron-donating group (Me), electron-withdrawing groups (NO_2_, CN) and halogens (F, Cl, Br) also reacted smoothly to achieve the desired products **6b**–**6g** in high yields (Table 3, entries 2–7). In addition, the reactions of 1-tetralones carrying electron-donating group (MeO), halogen (Br) and electron-withdrawing group (NO_2_) on the benzene ring afforded the corresponding products **6h**–**6j** in 81%–88% yields (Table 3, entries 8–10). Pleasingly, high yields were also obtained when 1-indanone and 4-chromanone were employed as substrates (Table 3, entries 11 and 12), these findings further broadened the substrate scope of this methodology. It should be noted that the structures of compounds **4** and **6** were confirmed by ^1^H-NMR (see Supplementary Files), ^13^C-NMR (see Supplementary Files), Low-resolution mass (LRMS) and high-resolution mass (HRMS).

## 3. Materials and Methods

### 3.1. General Information

The reagents were purchased from commercial suppliers and used without further purification. Analytical thin-layer chromatography (TLC) was performed on HSGF 254 (0.15–0.2 mm thickness), visualized by irradiation with UV light (254 nm). Column chromatography was performed using silica gel FCP 200–300. Melting points were measured with a micro melting point apparatus. Nuclear magnetic resonance spectra were recorded on a Brucker AMX-300 or 400 or 500 MHz instrument [TMS (Tetramethylsilane) as IS (Internal Standard)]. Chemical shifts were reported in parts per million (ppm, δ) downfield from tetramethylsilane. Proton coupling patterns were described as singlet (s), doublet (d), triplet (t), quartet (q), multiplet (m), and broad (br). Low and high-resolution mass (LRMS and HRMS) were measured by the EI (Electron Ionization) method with a Tsou-EI mass spectrometer. All the microwave-assisted reactions were performed in sealed tubes (capacity 10 mL) under a nitrogen atmosphere under a microwave heating system (CEM Discover) at the specified temperature. A feedback mechanism was involved in the temperature control system, and the reaction temperature which could be read from the temperature display screen was real-time monitored. It should be noted that a fixed power (30 W) was found to be appropriate to achieve the reaction temperature (90 °C) without overheating since a higher power led to the loss of efficacy of the temperature control system which resulted in overheating.

### 3.2. General Procedure for the Synthesis of 3,4-Dihydropyrimidin-2(1H)-thiones (***4***)

A high-pressure microwave vessel (capacity 10 mL) was loaded with ketones (0.5 mmol), benzaldehydes (0.5 mmol), thiourea (0.75 mmol), FeCl_3_∙6H_2_O (0.05 mmol) and TMSBr (0.5 mmol) in CH_3_CN (3.0 mL). The vessel was degassed, refilled with nitrogen, and sealed. Then the mixture was heated to 90 °C for 2 h under microwave irradiation using a CEM Discover (fixed power, 30 W). After cooling, the solids which had precipitated out were separated by filtration, and the solids obtained were washed with CH_3_CN to give the desired products **4**.

*4-Phenyl-3,4,5,6-tetrahydrobenzo[h]quinazoline-2(1H)-thione* (**4a**): White solid (128.7 mg, 88%), m.p. 246–248 °C. ^1^H-NMR (500 MHz, DMSO) δ 9.76 (s, 1H), 9.10 (s, 1H), 7.69 (d, *J* = 7.1 Hz, 1H), 7.39–7.35 (m, 2H), 7.34–7.28 (m, 3H), 7.24–7.16 (m, 2H), 7.15 (d, *J* = 6.6 Hz, 1H), 4.95 (s, 1H), 2.77–2.65 (m, 1H), 2.61–2.52 (m, 1H), 2.22–2.12 (m, 1H), 1.88–1.77 (m, 1H). ^13^C-NMR (126 MHz, DMSO) δ 174.28, 142.90, 135.45, 128.75, 127.98, 127.81, 127.75, 127.64, 127.02, 126.69, 126.38, 121.71, 111.23, 58.51, 27.37, 23.65. LRMS (EI): 292 (M^+^); HRMS (EI) calcd. for C_18_H_16_N_2_S (M^+^) 292.1034, found: 292.1031.

*4-p-Tolyl-3,4,5,6-tetrahydrobenzo[h]quinazoline-2(1H)-thione* (**4b**): White solid (131.3 mg, 86%), m.p. 230–232 °C. ^1^H-NMR (400 MHz, DMSO) δ 9.74 (s, 1H), 9.04 (s, 1H), 7.67 (d, *J* = 6.4 Hz, 1H), 7.23–7.15 (m, 7H), 4.90 (s, 1H), 2.71 (dt, *J* = 15.4, 7.6 Hz, 1H), 2.64–2.53 (m, 1H), 2.28 (s, 3H), 2.23–2.10 (m, 1H), 1.89–1.77 (m, 1H). ^13^C-NMR (126 MHz, DMSO) δ 174.15, 139.99, 137.20, 135.43, 129.25, 127.78, 127.75, 127.61, 126.96, 126.59, 126.35, 121.67, 111.37, 58.21, 27.36, 23.62, 20.75. LRMS (EI): 306 (M^+^); HRMS (EI) calcd. for C_19_H_18_N_2_S (M^+^) 306.1191, found: 306.1192.

*4-(3,4,5-Trimethoxyphenyl)-3,4,5,6-tetrahydrobenzo[h]quinazoline-2(1H)-thione* (**4c**): White solid (158.6 mg, 83%), m.p. 224–226 °C. ^1^H-NMR (400 MHz, DMSO) δ 9.79 (s, 1H), 9.01 (s, 1H), 7.75–7.53 (m, 1H), 7.31–7.09 (m, 3H), 6.64 (s, 2H), 4.92 (s, 1H), 3.74 (s, 6H), 3.65 (s, 3H), 2.80–2.70 (m, 1H), 2.70–2.58 (m, 1H), 2.29–2.12 (m, 1H), 2.04–1.84 (m, 1H). ^13^C-NMR (126 MHz, DMSO) δ 174.21, 152.99, 138.30, 137.12, 135.51, 127.76, 127.73, 127.60, 126.92, 126.32, 121.65, 111.01, 104.22, 59.99, 58.43, 55.86, 27.39, 23.59. LRMS (EI): 382 (M^+^); HRMS (EI) calcd. for C_21_H_22_N_2_O_3_S (M^+^) 382.1351, found: 382.1349.

*4-O-tolyl-3,4,5,6-tetrahydrobenzo[h]quinazoline-2(1H)-thione* (**4d**): White solid (122.9 mg, 80%), m.p. 241–242 °C. ^1^H-NMR (400 MHz, DMSO) δ 9.72 (s, 1H), 8.97 (s, 1H), 7.70 (dd, *J* = 8.3, 6.4 Hz, 1H), 7.33–7.07 (m, 7H), 5.25 (d, *J* = 2.0 Hz, 1H), 2.76–2.64 (m, 1H), 2.60–2.52 (m, 1H), 2.41 (s, 3H), 2.18–1.99 (m, 1H), 1.77–1.59 (m, 1H). ^13^C-NMR (126 MHz, DMSO) δ 173.94, 140.83, 135.64, 135.41, 130.60, 128.42, 127.85, 127.71, 127.68, 127.56, 126.74, 126.59, 126.33, 121.62, 111.15, 55.55, 27.28, 23.31, 18.83. LRMS (EI): 306 (M^+^); HRMS (EI) calcd. for C_19_H_18_N_2_S (M^+^) 306.1191, found: 306.1193.

*4-(3-Nitrilephenyl)-3,4,5,6-tetrahydrobenzo[h]quinazoline-2(1H)-thione* (**4e**): White solid (144.1 mg, 91%), m.p. 243–244 °C. ^1^H-NMR (400 MHz, DMSO) δ 9.90 (s, 1H), 9.12 (s, 1H), 7.83–7.78 (m, 1H), 7.73 (s, 1H), 7.72–7.59 (m, 3H), 7.26–7.19 (m, 2H), 7.19–7.13 (m, 1H), 5.09 (s, 1H), 2.78–2.67 (m, 1H), 2.66–2.54 (m, 1H), 2.26–2.14 (m, 1H), 1.90–1.79 (m, 1H). ^13^C-NMR (126 MHz, DMSO) δ 174.51, 144.24, 135.50, 131.83, 131.76, 130.51, 130.21, 127.94, 127.61, 127.47, 127.21, 126.31, 121.81, 118.63, 111.50, 110.19, 57.53, 27.22, 23.34. LRMS (EI): 317 (M^+^); HRMS (EI) calcd. for C_19_H_15_N_3_S (M^+^) 317.0987, found: 317.0979.

*methyl 4-(2-Thioxo-1,2,3,4,5,6-hexahydrobenzo[h]quinazolin-4-yl)benzoate* (**4f**): White solid (133.8 mg, 76%), m.p. 188–190 °C. ^1^H-NMR (400 MHz, DMSO) δ 9.86 (s, 1H), 9.17 (s, 1H), 7.98 (d, *J* = 8.1 Hz, 2H), 7.69 (d, *J* = 6.3 Hz, 1H), 7.47 (d, *J* = 8.1 Hz, 2H), 7.31–7.07 (m, 3H), 5.08 (s, 1H), 3.84 (s, 3H), 2.77–2.67 (m, 1H), 2.63–2.53 (m, 1H), 2.27–2.10 (m, 1H), 1.90–1.72 (m, 1H). ^13^C-NMR (126 MHz, DMSO) δ 174.49, 165.99, 147.92, 135.49, 129.73, 129.18, 127.95, 127.66, 127.61, 127.37, 126.99, 126.38, 121.79, 110.49, 58.13, 52.24, 27.31, 23.52. LRMS (EI): 350 (M^+^); HRMS (EI) calcd. for C_20_H_18_N_2_O_2_S (M^+^) 350.1089, found: 350.1085.

*4-(4-Fluorophenyl)-3,4,5,6-tetrahydrobenzo[h]quinazoline-2(1H)-thione* (**4g**): White solid (138.3 mg, 89%), m.p. 242–243 °C. ^1^H-NMR (400 MHz, DMSO) δ 9.80 (s, 1H), 9.10 (s, 1H), 7.68 (d, *J* = 6.4 Hz, 1H), 7.38–7.32 (m, 2H), 7.24–7.19 (m, 4H), 7.18–7.13 (m, 1H), 4.99 (s, 1H), 2.78–2.66 (m, 1H), 2.64–2.53 (m, 1H), 2.28–2.03 (m, 1H), 1.90–1.72 (m, 1H). ^13^C-NMR (126 MHz, DMSO) δ 174.23, 161.77 (d, *J*_C–F_ = 243.9 Hz), 139.13, 135.48, 129.07 (d, *J*_C–F_ = 8.3 Hz), 127.86, 127.68, 127.64, 126.82, 126.37, 121.76, 115.55 (d, *J*_C–F_ = 21.4 Hz), 111.05, 57.66, 27.34, 23.55. LRMS (EI): 310 (M^+^); HRMS (EI) calcd. for C_18_H_15_FN_2_S (M^+^) 310.0940, found: 310.0933.

*4-(4-Chlorophenyl)-3,4,5,6-tetrahydrobenzo[h]quinazoline-2(1H)-thione* (**4h**): White solid (146.8 mg, 90%), m.p. 226–228 °C. ^1^H-NMR (400 MHz, DMSO) δ 9.82 (s, 1H), 9.11 (s, 1H), 7.72–7.64 (m, 1H), 7.46 (d, *J* = 8.4 Hz, 2H), 7.33 (d, *J* = 8.7 Hz, 2H), 7.23–7.18 (m, 2H), 7.07 (s, 1H), 4.99 (s, 1H), 2.78–2.66 (m, 1H), 2.67–2.54 (m, 1H), 2.24–2.12 (m, 1H), 1.88–1.75 (m, 1H). ^13^C-NMR (126 MHz, DMSO) δ 174.26, 141.74, 135.43, 132.45, 128.84, 128.70, 127.83, 127.58, 126.87, 126.31, 121.72, 110.69, 57.64, 27.26, 23.46. LRMS (EI): 326 (M^+^, Cl^35^), 328 (M^+^, Cl^37^); HRMS (EI) calcd. for C_18_H_15_ClN_2_S (M^+^) 326.0644, found: 326.0636.

*4-(4-Bromophenyl)-3,4,5,6-tetrahydrobenzo[h]quinazoline-2(1H)-thione* (**4i**): White solid (166.5 mg, 90%), m.p. 229–230 °C. ^1^H-NMR (400 MHz, DMSO) δ 9.82 (s, 1H), 9.12 (s, 1H), 7.73–7.65 (m, 1H), 7.59 (d, *J* = 8.3 Hz, 2H), 7.27 (d, *J* = 8.3 Hz, 2H), 7.24–7.13 (m, 3H), 4.97 (s, 1H), 2.77–2.65 (m, 1H), 2.64–2.54 (m, 1H), 2.27–2.09 (m, 1H), 1.89–1.74 (m, 1H). ^13^C-NMR (126 MHz, DMSO) δ 174.27, 142.14, 135.44, 131.63, 129.20, 127.85, 127.59, 126.89, 126.32, 121.73, 121.05, 110.64, 57.72, 27.27, 23.46. LRMS (EI): 370 (M^+^, Br^79^), 372 (M^+^, Br^81^); HRMS (EI) calcd. for C_18_H_15_BrN_2_S (M^+^) 370.0139, found: 370.0134.

*8-Methoxy-4-phenyl-3,4,5,6-tetrahydrobenzo[h]quinazoline-2(1H)-thione* (**4j**): White solid (135.0 mg, 84%), m.p. 247–248 °C. ^1^H-NMR (400 MHz, DMSO) δ 9.71 (s, 1H), 9.06 (s, 1H), 7.73–7.55 (m, 1H), 7.38 (dd, *J* = 8.9, 5.7 Hz, 2H), 7.34–7.27 (m, 3H), 6.82–6.69 (m, 2H), 4.92 (s, 1H), 3.74 (s, 3H), 2.76–2.63 (m, 1H), 2.63–2.53 (m, 1H), 2.22–2.10 (m, 1H), 1.87–1.72 (m, 1H). ^13^C-NMR (101 MHz, DMSO) δ 174.20, 158.91, 143.10, 137.53, 128.74, 127.93, 127.01, 126.53, 123.12, 120.63, 113.97, 110.85, 108.56, 58.51, 55.18, 27.78, 23.62. LRMS (EI): 322 (M^+^); HRMS (EI) calcd. for C_19_H_18_N_2_OS (M^+^) 322.1140, found: 322.1139.

*9-Bromo-4-phenyl-3,4,5,6-tetrahydrobenzo[h]quinazoline-2(1H)-thione* (**4k**): White solid (159.1 mg, 86%), m.p. 225–226 °C. ^1^H-NMR (400 MHz, DMSO) δ 9.97 (s, 1H), 9.12 (s, 1H), 7.96 (s, 1H), 7.42–7.35 (m, 3H), 7.35–7.28 (m, 3H), 7.12 (d, *J* = 8.0 Hz, 1H), 4.96 (s, 1H), 2.74–2.63 (m, 1H), 2.60–2.52 (m, 1H), 2.28–2.09 (m, 1H), 1.93–1.74 (m, 1H). ^13^C-NMR (101 MHz, DMSO) δ 174.38, 142.67, 134.76, 130.27, 129.94, 129.58, 128.81, 128.07, 127.03, 125.93, 124.67, 119.60, 112.85, 58.44, 26.74, 23.51. LRMS (EI): 370 (M^+^, Br^79^), 372 (M^+^, Br^81^); HRMS (EI) calcd. for C_18_H_15_BrN_2_S (M^+^) 370.0139, found: 370.0145.

*9-Nitro-4-phenyl-3,4,5,6-tetrahydrobenzo[h]quinazoline-2(1H)-thione* (**4l**): White solid (154.8 mg, 92%), m.p. 228–229 °C. ^1^H-NMR (400 MHz, DMSO) δ 10.36 (s, 1H), 9.18 (s, 1H), 8.63 (d, *J* = 2.3 Hz, 1H), 8.08 (dd, *J* = 8.2, 2.3 Hz, 1H), 7.45 (d, *J* = 8.3 Hz, 1H), 7.43–7.37 (m, 2H), 7.36–7.31 (m, 3H), 5.00 (d, *J* = 2.4 Hz, 1H), 2.93–2.81 (m, 1H), 2.72 (ddd, *J* = 16.0, 9.0, 6.9 Hz, 1H), 2.37–2.15 (m, 1H), 2.02–1.78 (m, 1H). ^13^C-NMR (101 MHz, DMSO) δ 174.53, 146.49, 143.72, 142.50, 129.21, 128.81, 128.09, 127.06, 125.73, 122.63, 116.89, 113.98, 58.33, 27.34, 23.02. LRMS (EI): 337 (M^+^); HRMS (EI) calcd. for C_18_H_15_N_3_O_2_S (M^+^) 337.0885, found: 337.0889.

*4-Phenyl-3,4-dihydro-1H-indeno[1,2-d]pyrimidine-2(5H)-thione* (**4m**): White solid (123.4 mg, 89%), m.p. 199–201 °C. ^1^H-NMR (400 MHz, DMSO) δ 10.82 (s, 1H), 9.07 (s, 1H), 7.96–7.71 (m, 1H), 7.42–7.24 (m, 7H), 7.22–7.11 (m, 1H), 5.51 (s, 1H), 3.33 (d, *J* = 23.8 Hz, 1H), 2.88 (d, *J* = 23.2 Hz, 1H). ^13^C-NMR (126 MHz, DMSO) δ 174.24, 143.42, 142.26, 136.48, 132.95, 128.76, 127.75, 126.57, 126.40, 125.45, 124.09, 118.84, 115.34, 57.56, 34.92. LRMS (EI): 278 (M^+^); HRMS (EI) calcd. for C_17_H_14_N_2_S (M^+^) 278.0878, found: 278.0877.

### 3.3. General Procedure for the Synthesis of 3,4-Dihydropyrimidin-2(1H)-ones (***6***)

A high-pressure microwave vessel (capacity 10 mL) was loaded with ketones (0.5 mmol), benzaldehydes (0.5 mmol), urea (0.75 mmol), FeCl_3_∙6H_2_O (0.05 mmol) and TMSBr (0.5 mmol) in CH_3_CN (3.0 mL). The vessel was degassed, refilled with nitrogen, and sealed. Then the mixture was heated to 90 °C for 2 h under microwave irradiation using a CEM Discover (fixed power, 30 W). After cooling, the solids which had precipitated out were separated by filtration, and the solids obtained were washed with CH_3_CN to give the desired products **6**.

*4-Phenyl-3,4,5,6-tetrahydrobenzo[h]quinazolin-2(1H)-one* (**6a**): White solid (124.1 mg, 90%), m.p. 270–272 °C. ^1^H-NMR (400 MHz, DMSO) δ 8.60 (s, 1H), 7.58 (dd, *J* = 13.4, 6.3 Hz, 1H), 7.39–7.30 (m, 5H), 7.30–7.24 (m, 1H), 7.25–7.12 (m, 3H), 4.94 (s, 1H), 2.75–2.65 (m, 1H), 2.62–2.52 (m, 1H), 2.19–2.03 (m, 1H), 1.84–1.65 (m, 1H). ^13^C-NMR (101 MHz, DMSO) δ 153.49, 144.20, 135.47, 128.85, 128.65, 127.72, 127.67, 127.54, 127.52, 126.95, 126.40, 121.27, 108.16, 59.16, 27.66, 23.58. LRMS (EI): 276 (M^+^); HRMS (EI) calcd. for C_18_H_16_N_2_O (M^+^) 276.1263, found: 276.1260.

*4-M-tolyl-3,4,5,6-tetrahydrobenzo[h]quinazolin-2(1H)-one* (**6b**): White solid (124.9 mg, 86%), m.p. 273–275 °C. ^1^H-NMR (400 MHz, DMSO) δ 8.54 (s, 1H), 7.57 (d, *J* = 6.8 Hz, 1H), 7.27–7.17 (m, 4H), 7.16–7.08 (m, 4H), 4.89 (s, 1H), 2.75–2.65 (m, 1H), 2.62–2.52 (m, 1H), 2.29 (s, 3H), 2.19–2.02 (m, 1H), 1.84–1.67 (m, 1H). ^13^C-NMR (101 MHz, DMSO) δ 153.41, 144.19, 137.71, 135.47, 128.87, 128.54, 128.31, 127.63, 127.54, 127.49, 127.47, 126.39, 124.14, 121.25, 108.17, 59.18, 27.66, 23.56, 21.16. LRMS (EI): 290 (M^+^); HRMS (EI) calcd. for C_19_H_18_N_2_O (M^+^) 290.1419, found: 276.1412.

*4-(4-Nitrophenyl)-3,4,5,6-tetrahydrobenzo[h]quinazolin-2(1H)-one* (**6c**): White solid (146.1 mg, 91%), m.p. 214–216 °C. ^1^H-NMR (400 MHz, DMSO) δ 8.71 (s, 1H), 8.24 (d, *J* = 8.7 Hz, 2H), 7.64–7.56 (m, 3H), 7.46 (s, 1H), 7.24–7.11 (m, 3H), 5.14 (s, 1H), 2.77–2.65 (m, 1H), 2.64–2.52 (m, 1H), 2.22–2.09 (m, 1H), 1.81–1.68 (m, 1H). ^13^C-NMR (126 MHz, DMSO) δ 153.18, 146.62, 143.71, 143.66, 130.27, 128.66, 127.74, 126.93, 126.78, 122.34, 116.19, 110.97, 58.94, 27.59, 22.92. LRMS (EI): 321 (M^+^); HRMS (EI) calcd. for C_18_H_15_N_3_O_3_ (M^+^) 321.1113, found: 321.1114.

*3-(2-Oxo-1,2,3,4,5,6-hexahydrobenzo[h]quinazolin-4-yl)benzonitrile* (**6d**): White solid (127.1 mg, 84%), m.p. 286–287 °C. ^1^H-NMR (400 MHz, DMSO) δ 8.70 (s, 1H), 7.81–7.73 (m, 2H), 7.68 (s, 1H), 7.65–7.56 (m, 2H), 7.41 (s, 1H), 7.26–7.10 (m, 3H), 5.07 (s, 1H), 2.78–2.66 (m, 1H), 2.64–2.54 (m, 1H), 2.20–2.08 (m, 1H), 1.81–1.69 (m, 1H). ^13^C-NMR (126 MHz, DMSO) δ 153.33, 145.69, 135.58, 131.95, 131.62, 130.59, 130.22, 128.64, 128.38, 127.78, 127.64, 126.46, 121.47, 118.86, 111.46, 107.09, 58.35, 27.62, 23.34. LRMS (EI): 301 (M^+^); HRMS (EI) calcd. for C_19_H_15_N_3_O (M^+^) 301.1215, found: 301.1210.

*4-(4-Fluorophenyl)-3,4,5,6-tetrahydrobenzo[h]quinazolin-2(1H)-one* (**6e**): White solid (120.9 mg, 82%), m.p. 209–210 °C. ^1^H-NMR (400 MHz, DMSO) δ 8.58 (s, 1H), 7.66–7.51 (m, 1H), 7.40–7.32 (m, 2H), 7.30 (s, 1H), 7.25–7.10 (m, 5H), 4.97 (s, 1H), 2.76–2.66 (m, 1H), 2.63–2.53 (m, 1H), 2.19–1.99 (m, 1H), 1.81–1.67 (m, 1H). ^13^C-NMR (101 MHz, DMSO) δ 161.58 (d, *J*_C–F_ = 243.1 Hz), 153.27 (s), 140.43 (s), 135.45 (s), 128.90 (d, *J*_C–F_ = 8.2 Hz), 128.75 (s), 127.82 (s), 127.51 (s), 126.35 (s), 121.29 (s), 115.35 (d, *J*_C–F_ = 21.4 Hz), 107.89 (s), 58.30 (s), 27.61 (s), 23.45 (s). LRMS (EI): 294 (M^+^); HRMS (EI) calcd. for C_18_H_15_FN_2_O (M^+^) 294.1168, found: 294.1168.

*4-(3-Chlorophenyl)-3,4,5,6-tetrahydrobenzo[h]quinazolin-2(1H)-one* (**6f**): White solid (140.1 mg, 90%), m.p. 279–280 °C. ^1^H-NMR (400 MHz, DMSO) δ 8.65 (s, 1H), 7.59 (d, *J* = 6.6 Hz, 1H), 7.42-7.33 (m, 4H), 7.30 (d, *J* = 7.4 Hz, 1H), 7.24–7.12 (m, 3H), 4.94 (s, 1H), 2.77–2.65 (m, 1H), 2.63–2.53 (m, 1H), 2.21–2.03 (m, 1H), 1.87–1.70 (m, 1H). ^13^C-NMR (101 MHz, DMSO) δ 153.33, 146.67, 135.50, 133.23, 130.68, 128.67, 128.11, 127.66, 127.62, 127.59, 126.77, 126.41, 125.61, 121.36, 107.46, 58.50, 27.62, 23.42. LRMS (EI): 310 (M^+^, Cl^35^), 312 (M^+^, Cl^37^); HRMS (EI) calcd. for C_18_H_15_ClN_2_O (M^+^) 310.0873, found: 310.0864.

*4-(2-Bromophenyl)-3,4,5,6-tetrahydrobenzo[h]quinazolin-2(1H)-one* (**6g**): White solid (142.7 mg, 80%), m.p. 271–273 °C. ^1^H-NMR (400 MHz, DMSO) δ 8.67 (s, 1H), 7.63–7.57 (m, 2H), 7.49–7.39 (m, 2H), 7.33 (s, 1H), 7.27–7.16 (m, 3H), 7.16–7.11 (m, 1H), 5.54–5.38 (m, 1H), 2.75–2.64 (m, 1H), 2.59–2.52 (m, 1H), 2.22–2.01 (m, 1H), 1.81–1.58 (m, 1H). ^13^C-NMR (101 MHz, DMSO) δ 153.15, 142.77, 135.53, 132.70, 130.12, 129.79, 128.72, 128.64, 128.22, 127.66, 127.55, 126.42, 121.95, 121.38, 107.18, 58.35, 27.55, 22.97. LRMS (EI): 354 (M^+^, Br^79^), 356 (M^+^, Br^81^); HRMS (EI) calcd. for C_18_H_15_BrN_2_O (M^+^) 354.0368, found: 354.0366.

*8-Methoxy-4-phenyl-3,4,5,6-tetrahydrobenzo[h]quinazolin-2(1H)-one* (**6h**): White solid (124.4 mg, 81%), m.p. 247–249 °C. ^1^H-NMR (400 MHz, DMSO) δ 8.54 (s, 1H), 7.54 (d, *J* = 9.2 Hz, 1H), 7.39–7.30 (m, 4H), 7.30–7.23 (m, 2H), 6.82–6.67 (m, 2H), 4.90 (s, 1H), 3.74 (s, 3H), 2.75–2.63 (m, 1H), 2.60–2.52 (m, 1H), 2.15–2.03 (m, 1H), 1.80–1.68 (m, 1H). ^13^C-NMR (101 MHz, DMSO) δ 158.66, 153.45, 144.37, 137.44, 128.57, 127.55, 127.50, 126.91, 122.59, 121.71, 113.81, 110.81, 105.43, 59.11, 55.10, 28.03, 23.50. LRMS (EI): 306 (M^+^); HRMS (EI) calcd. for C_19_H_18_N_2_O_2_ (M^+^) 306.1368, found: 306.1367.

*9-Bromo-4-phenyl-3,4,5,6-tetrahydrobenzo[h]quinazolin-2(1H)-one* (**6i**): White solid (147.2 mg, 83%), m.p. 277–279 °C. ^1^H-NMR (400 MHz, DMSO) δ 8.67 (s, 1H), 7.78 (s, 1H), 7.40–7.27 (m, 7H), 7.10 (d, *J* = 7.9 Hz, 1H), 4.90 (s, 1H), 2.72–2.61 (m, 1H), 2.59–2.52 (m, 1H), 2.24–2.05 (m, 1H), 1.89–1.65 (m, 1H). ^13^C-NMR (101 MHz, DMSO) δ 153.25, 143.94, 134.73, 131.04, 129.98, 129.48, 128.91, 128.68, 127.74, 126.95, 124.17, 119.62, 109.87, 59.06, 27.02, 23.40. LRMS (EI): 354 (M^+^, Br^79^), 356 (M^+^, Br^81^); HRMS (EI) calcd. for C_18_H_15_BrN_2_O (M^+^) 354.0368, found: 354.0371.

*9-Nitro-4-phenyl-3,4,5,6-tetrahydrobenzo[h]quinazolin-2(1H)-one* (**6j**): White solid (141.1 mg, 88%), m.p. 319–321 °C. ^1^H-NMR (400 MHz, DMSO) δ 8.99 (s, 1H), 8.48 (d, *J* = 2.2 Hz, 1H), 8.06 (dd, *J* = 8.2, 2.2 Hz, 1H), 7.45–7.40 (m, 1H), 7.40–7.31 (m, 5H), 7.32–7.26 (m, 1H), 4.97 (s, 1H), 2.84 (m, 1H), 2.77–2.64 (m, 1H), 2.27–2.14 (m, 1H), 1.81 (m, 1H). ^13^C-NMR (126 MHz, DMSO) δ 153.18, 146.62, 143.71, 143.66, 130.27, 128.66, 127.74, 126.93, 126.78, 122.34, 116.19, 110.97, 58.94, 27.59, 22.92. LRMS (EI): 321 (M^+^); HRMS (EI) calcd. for C_18_H_15_N_3_O_3_ (M^+^) 321.1113, found: 321.1108.

*4-Phenyl-3,4-dihydro-1H-indeno[1,2-d]pyrimidin-2(5H)-one* (**6k**): White solid (114.3 mg, 87%), m.p. 269–270 °C. ^1^H-NMR (400 MHz, DMSO) δ 9.45 (s, 1H), 7.62 (d, *J* = 7.5 Hz, 1H), 7.45–7.20 (m, 8H), 7.20–7.08 (m, 1H), 5.45 (s, 1H), 3.26 (d, *J* = 22.9 Hz, 1H), 2.78 (d, *J* = 22.8 Hz, 1H). ^13^C-NMR (101 MHz, DMSO) δ 153.38, 144.46, 142.76, 137.51, 134.82, 128.63, 127.42, 126.40, 126.23, 125.11, 123.95, 118.19, 112.24, 57.18, 34.66. LRMS (EI): 262 (M^+^); HRMS (EI) calcd. for C_17_H_14_N_2_O (M^+^) 262.1106, found: 262.1103.

*4-Phenyl-3,4-dihydro-1H-chromeno[4,3-d]pyrimidin-2(5H)-one* (**6l**): White solid (113.8 mg, 82%), m.p. 251–252 °C. ^1^H-NMR (400 MHz, DMSO) δ 8.91 (s, 1H), 7.63 (dd, *J* = 7.8, 1.3 Hz, 1H), 7.47 (s, 1H), 7.43–7.27 (m, 5H), 7.24–7.14 (m, 1H), 7.00–6.90 (m, 1H), 6.83–6.75 (m, 1H), 4.98 (s, 1H), 4.74–4.68 (m, 1H), 4.21 (d, *J* = 13.6 Hz, 1H). ^13^C-NMR (126 MHz, DMSO) δ 153.48, 152.99, 143.11, 129.61, 128.78, 127.90, 126.76, 125.17, 121.97, 121.22, 117.40, 115.83, 101.30, 64.80, 56.08. LRMS (EI): 278 (M^+^); HRMS (EI) calcd. for C_17_H_14_N_2_O_2_ (M^+^) 278.1055, found: 278.1049.

## 4. Conclusions

In conclusion, we have developed an efficient and practical approach to synthesize dihydropyrimidinones and dihydropyrimidinethiones through FeCl_3_∙6H_2_O/TMSBr-catalyzed three-component cyclocondensation under microwave heating. This protocol features high yields, broad substrate scope, short reaction time, mild reaction conditions, operational simplicity and easy work-up. These advantages demonstrate the great potential of this method for the synthesis of dihydropyrimidinones and dihydropyrimidinethiones. More importantly, our ongoing research has revealed that the simple derivatives of compounds **4** or **6** are found to be potential EV71 3C protein inhibitors, which are worthy of further investigation for the development of medical therapies for hand, foot and mouth disease (HFMD). We anticipate that these important heterocyclic compounds may find their potent pharmaceutical applications after further exploration.

## Figures and Tables

**Table 1:**
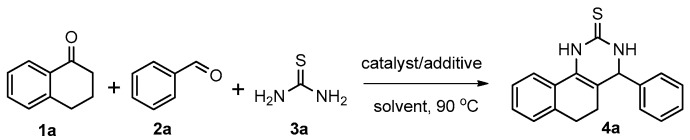
Optimization of the reaction conditions ^a^.

Entry	Catalyst	Additive	Solvent	Time	Yield (%) ^b^
1 ^c^	FeCl_3_∙6H_2_O	-	CH_3_CN	10 h	38
2	FeCl_3_∙6H_2_O	-	CH_3_CN	2 h	37
3	ZnCl_2_	-	CH_3_CN	2 h	trace
4	FeSO_4_∙7H_2_O	-	CH_3_CN	2 h	trace
5	CuBr_2_	-	CH_3_CN	2 h	16
6	AlCl_3_	-	CH_3_CN	2 h	18
7	FeCl_3_∙6H_2_O	BF_3_∙OEt_2_	CH_3_CN	2 h	43
8	FeCl_3_∙6H_2_O	BBr_3_	CH_3_CN	2 h	41
9	FeCl_3_∙6H_2_O	TMSOTf	CH_3_CN	2 h	65
10	FeCl_3_∙6H_2_O	TMSCl	CH_3_CN	2 h	82
11	FeCl_3_∙6H_2_O	TMSBr	CH_3_CN	2 h	88
12	FeCl_3_∙6H_2_O	TMSI	CH_3_CN	2 h	73
13	FeCl_3_∙6H_2_O	TMSBr	Toluene	2 h	44
14	FeCl_3_∙6H_2_O	TMSBr	THF	2 h	61
15	FeCl_3_∙6H_2_O	TMSBr	1,4-Dioxane	2 h	71
16	FeCl_3_∙6H_2_O	TMSBr	EtOH	2 h	84
17	FeCl_3_∙6H_2_O	TMSBr	neat	2 h	52
18 ^c^	FeCl_3_∙6H_2_O	TMSBr	CH_3_CN	8 h	80
19 ^c^	FeCl_3_∙6H_2_O	TMSBr	CH_3_CN	10 h	87

^a^ Unless noted, reactions were performed with **1a** (0.5 mmol), **2a** (0.5 mmol), **3a** (0.75 mmol), catalyst (0.05 mmol) and additive (0.5 mmol) in CH_3_CN (3.0 mL) at 90 °C under microwave irradiation (sealed vessel at fixed power, 30 W); ^b^ Isolated yield; ^c^ Heated with oil bath; TMSOTf = Trimethylsilyl trifluoromethanesulfonate, TMSCl = Chlorotrimethylsilane, TMSBr = Bromotrimethylsilane, TMSI = Iodotrimethylsilane.

**Table 2:**
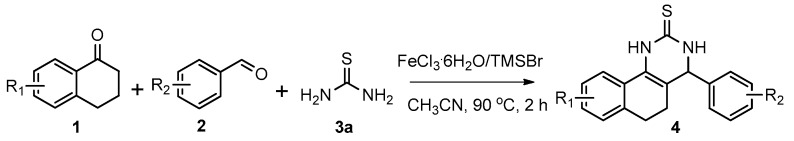
FeCl_3_∙6H_2_O/TMSBr catalyzed synthesis of 3,4-dihydropyrimidin-2(1*H*)-thiones ^a^.

Entry	Ketones (1)	Benzaldehydes (2)	Products (4)	Yield (%) ^b^
1	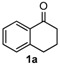	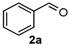	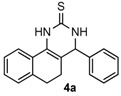	88
2	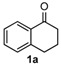	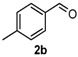	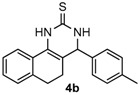	86
3	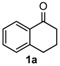	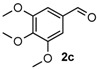	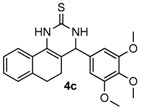	83
4	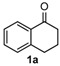	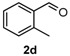	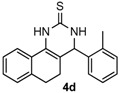	80
5	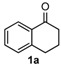	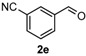	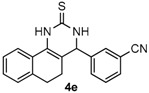	91
6	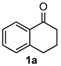	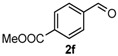	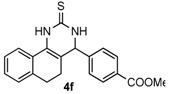	76
7	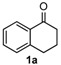	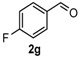	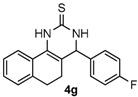	89
8	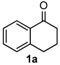	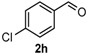	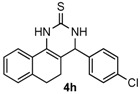	90
9	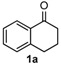	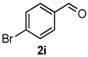	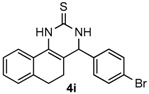	90
10	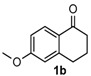	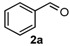	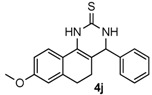	84
11	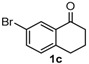	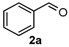	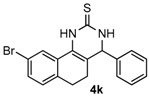	86
12	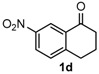	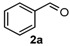	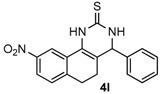	92
13	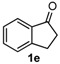	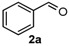	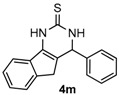	89

^a^ Reaction conditions: **1** (0.5 mmol), **2** (0.5 mmol), **3a** (0.75 mmol), FeCl_3_∙6H_2_O (0.05 mmol) and TMSBr (0.5 mmol) in CH_3_CN (3.0 mL) at 90 °C for 2 h under microwave irradiation (sealed vessel at fixed power, 30 W); ^b^ Isolated yield.

**Table 3:**
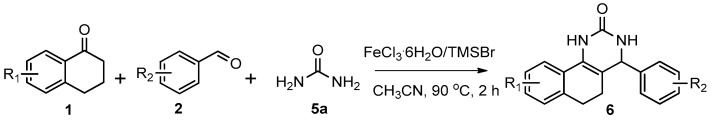
FeCl_3_∙6H_2_O/TMSBr-catalyzed synthesis of 3,4-dihydropyrimidin-2(1*H*)-ones ^a^.

Entry	Ketones (1)	Benzaldehydes (2)	Products (6)	Yield (%) ^b^
1	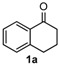	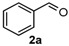	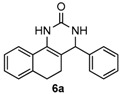	90
2	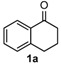	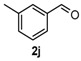	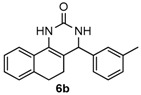	86
3	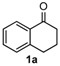	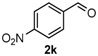	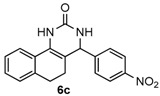	91
4	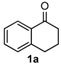	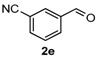	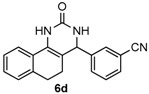	84
5	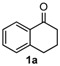	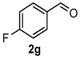	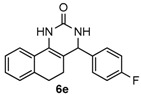	82
6	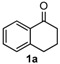	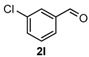	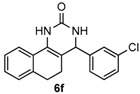	90
7	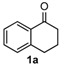	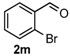	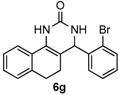	80
8	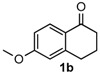	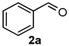	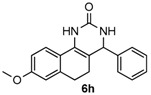	81
9	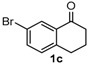	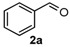	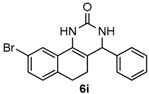	83
10	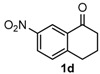	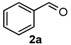	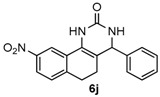	88
11	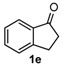	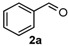	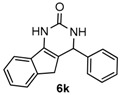	87
12	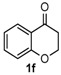	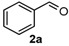	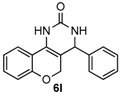	82

^a^ Reaction conditions: **1** (0.5 mmol), **2** (0.5 mmol), **5a** (0.75 mmol), FeCl_3_∙6H_2_O (0.05 mmol) and TMSBr (0.5 mmol) in CH_3_CN (3.0 mL) at 90 °C for 2 h under microwave irradiation (sealed vessel at fixed power, 30 W); ^b^ Isolated yield.

## Supplementary Materials

Supplementary Files (Copies of ^1^H and ^13^C-NMR spectra of compounds **4** and **6**) are available online.
