# Supplementary material for: FeCl3∙6H2O/TMSBr-Catalyzed Rapid Synthesis of Dihydropyrimidinones and Dihydropyrimidinethiones under Microwave Irradiation
Source: Molecules. 2017 Sep 11;22(9):1503. doi: 10.3390/molecules22091503 (PMC6151402; doi:10.3390/molecules22091503)

$FeCl_3 \cdot 6H_2O$/TMSBr Catalyzed Rapid Synthesis of Dihydropyrimidinones and Dihydropyrimidinethiones under Microwave Irradiation

Supporting Information

Fei Zhao *, Xiuwen Jia, Pinyi Li, Jingwei Zhao, Jun Huang, Honglian Li and Lin Li

Antibiotics Research and Re-evaluation Key Laboratory of Sichuan Province, Sichuan Industrial Institute of Antibiotics, Chengdu University, 168 Hua Guan Road, Chengdu 610052, P. R. China.

* Correspondence: zhaofei@cdu.edu.cn; Tel.: +86-187-8025-5276

Table of Contents

^{1}H and ^{13}C NMR Spectra……………………………………..……………..…...2-26

^{1}H and ^{13}CNMR Spectra

4-phenyl-3,4,5,6-tetrahydrobenzo[*h*]quinazoline-2(1*H*)-thione (4a)

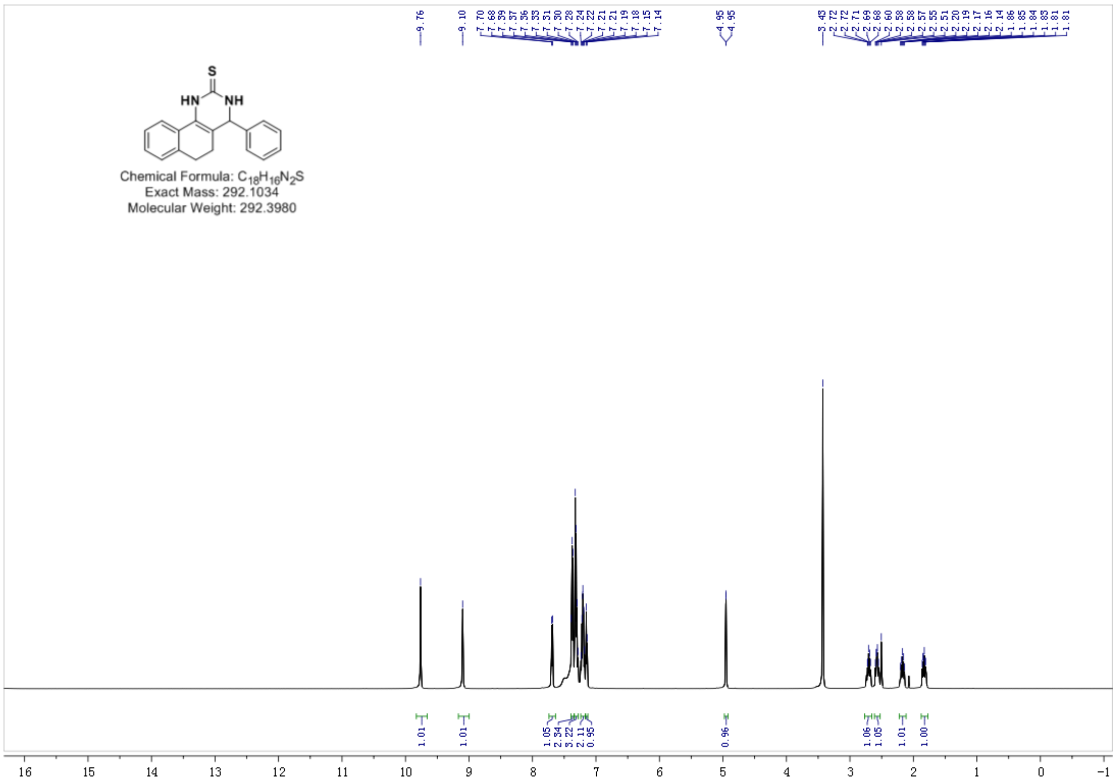

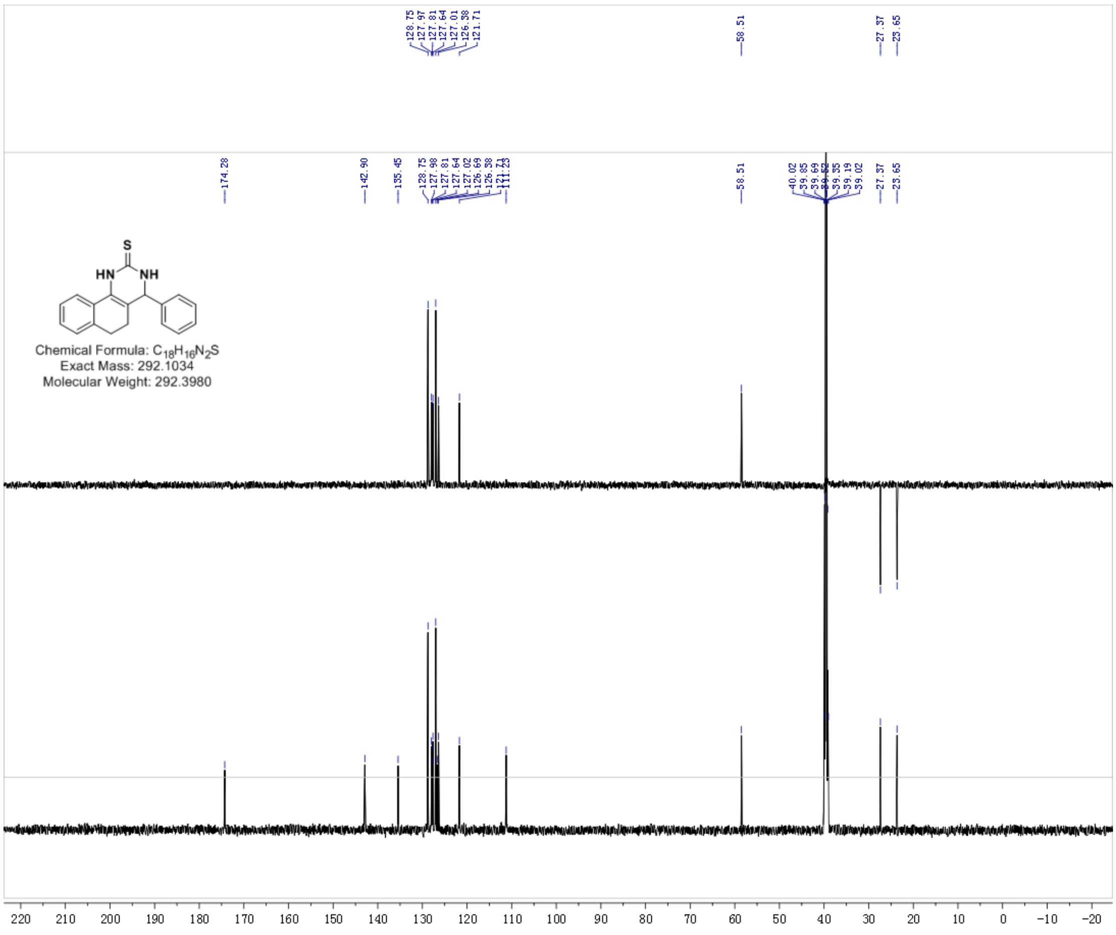

4-*p*-tolyl-3,4,5,6-tetrahydrobenzo[*h*]quinazoline-2(1*H*)-thione (4b)

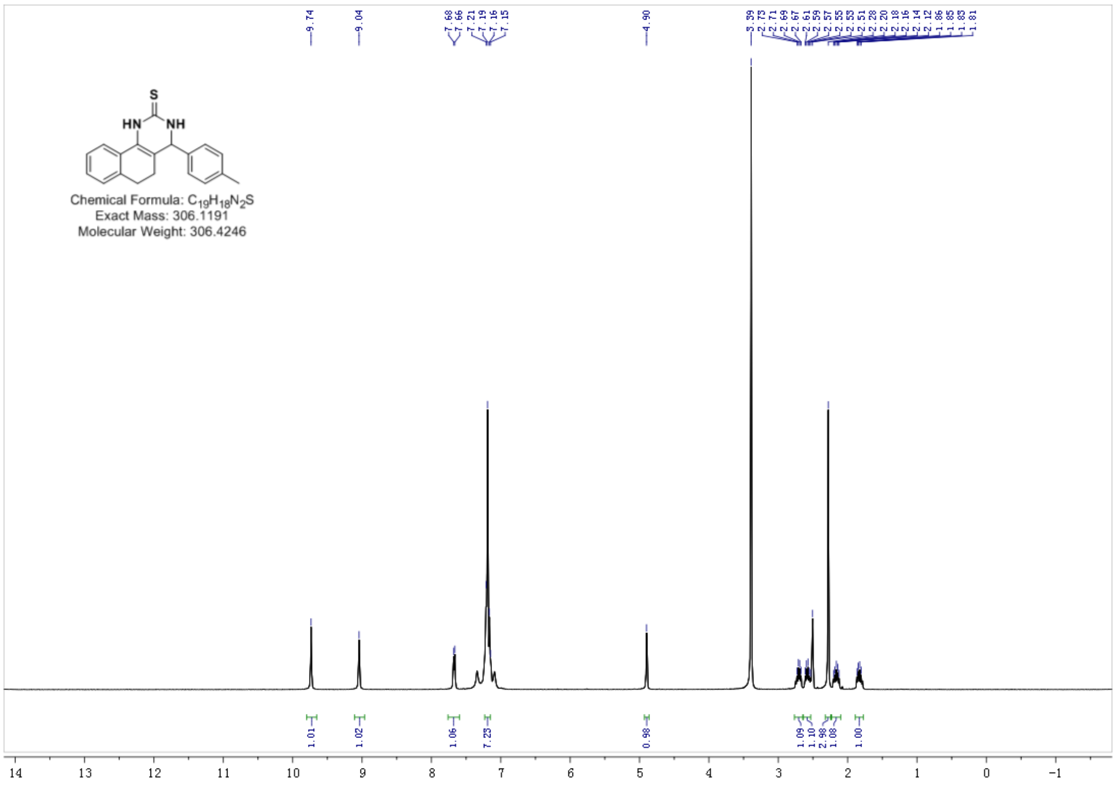

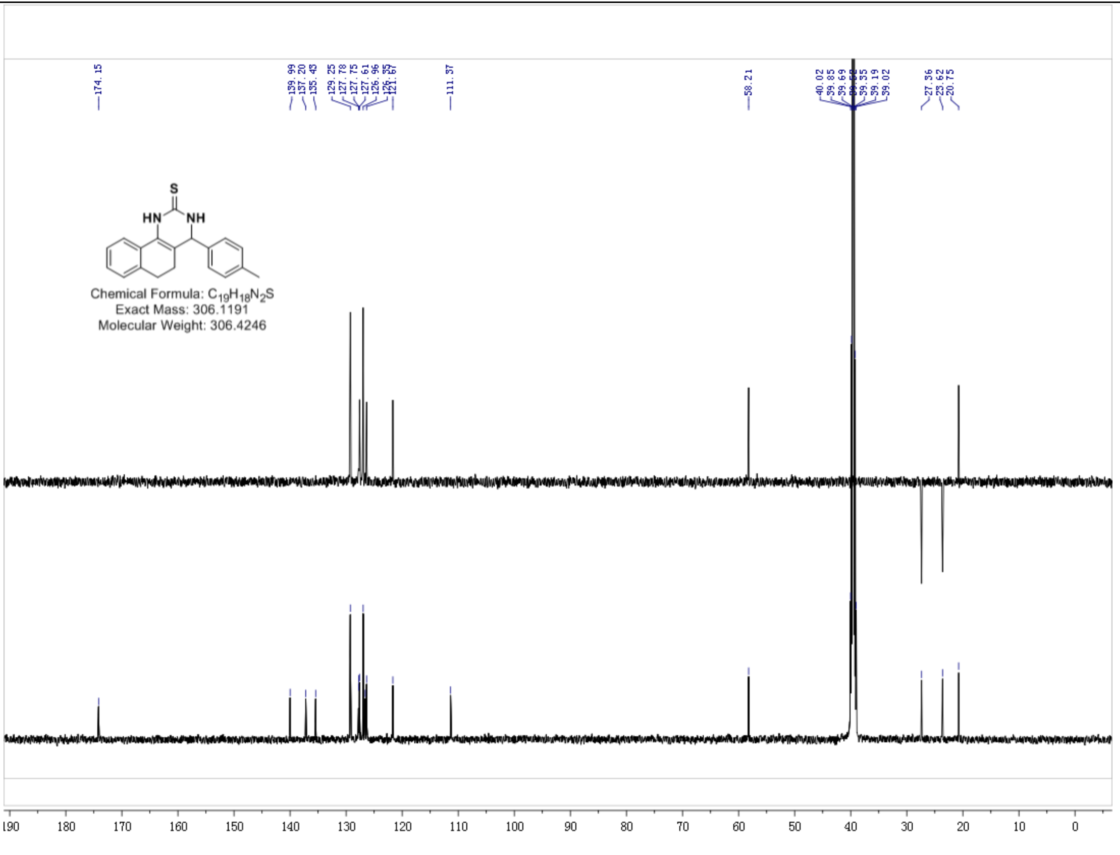

4-(3,4,5-trimethoxyphenyl)-3,4,5,6-tetrahydrobenzo[*h*]quinazoline-2(*1H*)-thione (4c)

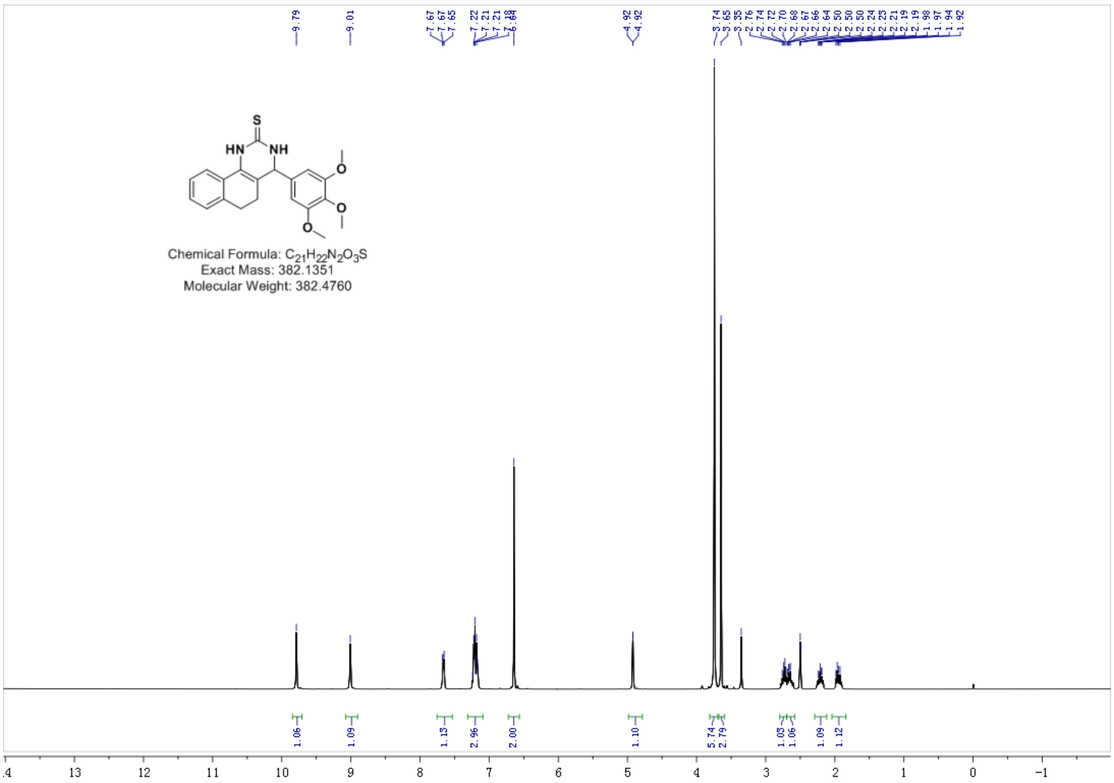

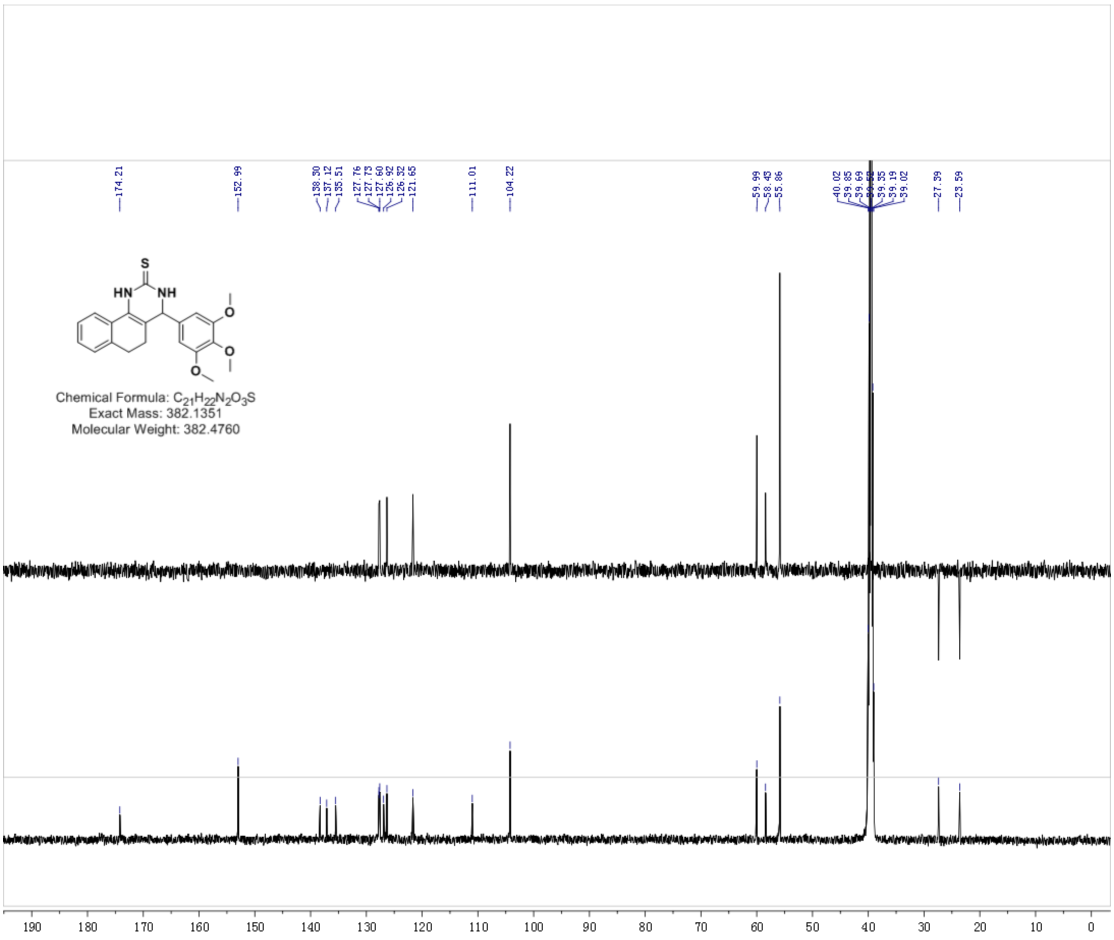

4-*o*-tolyl-3,4,5,6-tetrahydrobenzo[*h*]quinazoline-2(*1H*)-thione (4d)

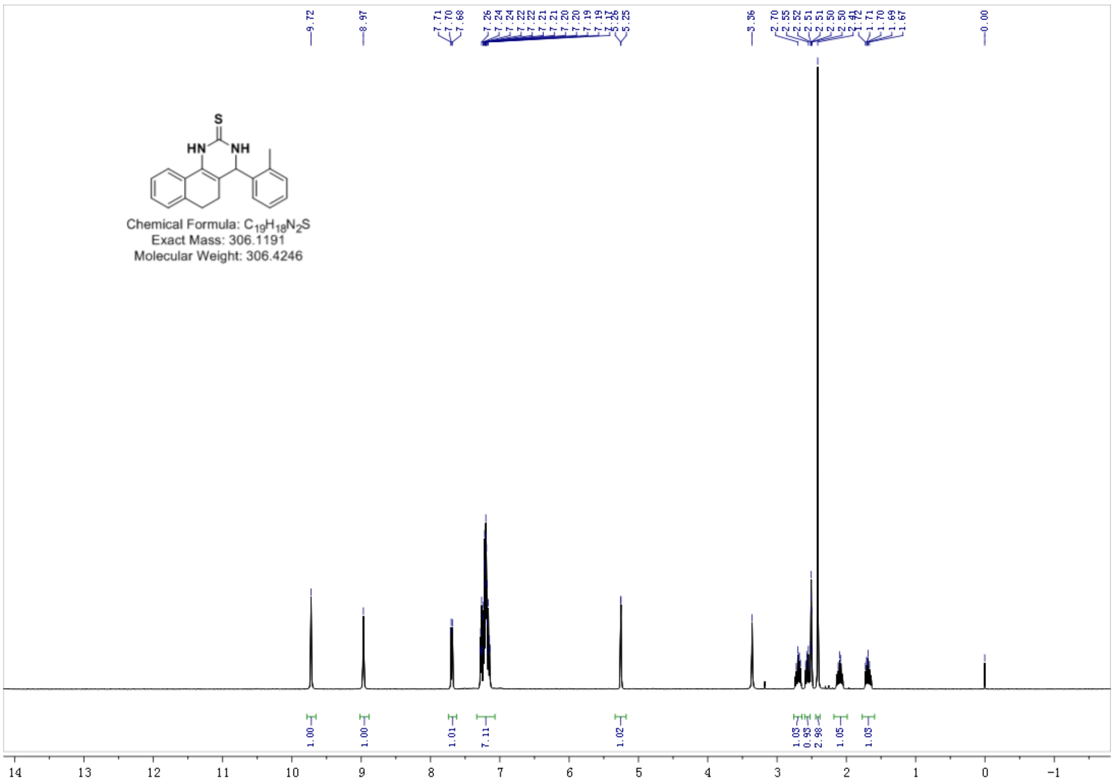

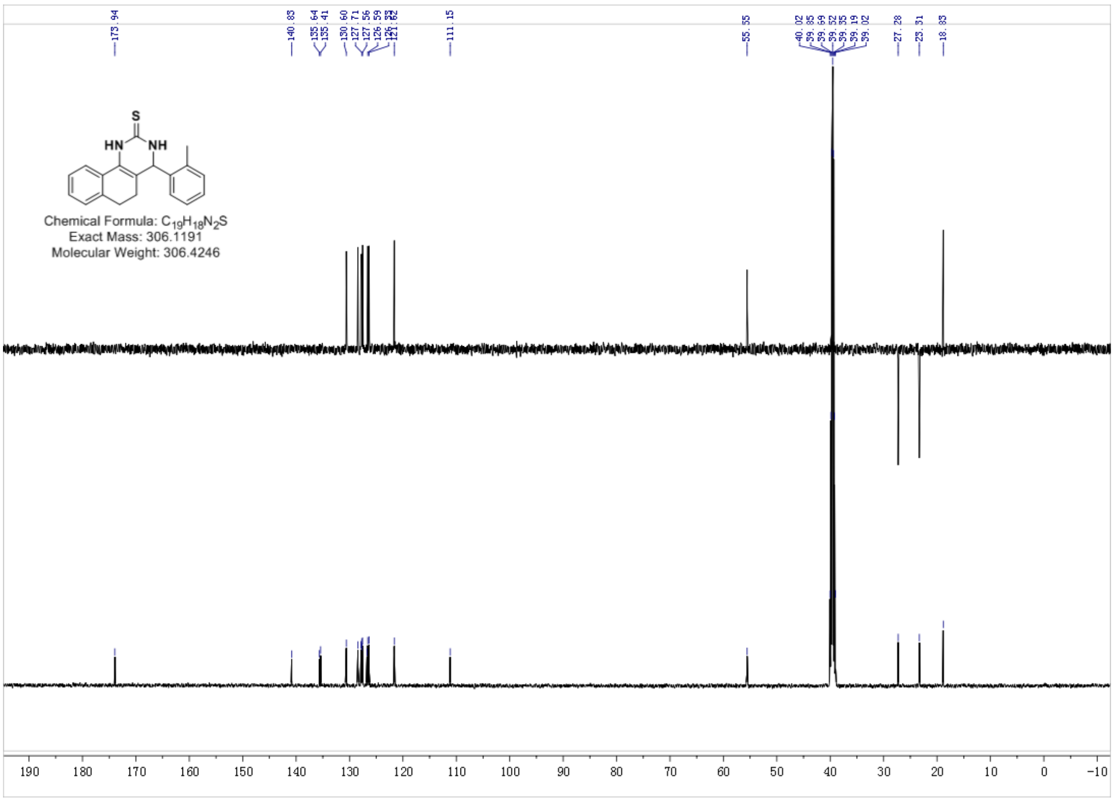

4-(3-nitrilephenyl)-3,4,5,6-tetrahydrobenzo[*h*]quinazoline-2(*1H*)-thione (4e)

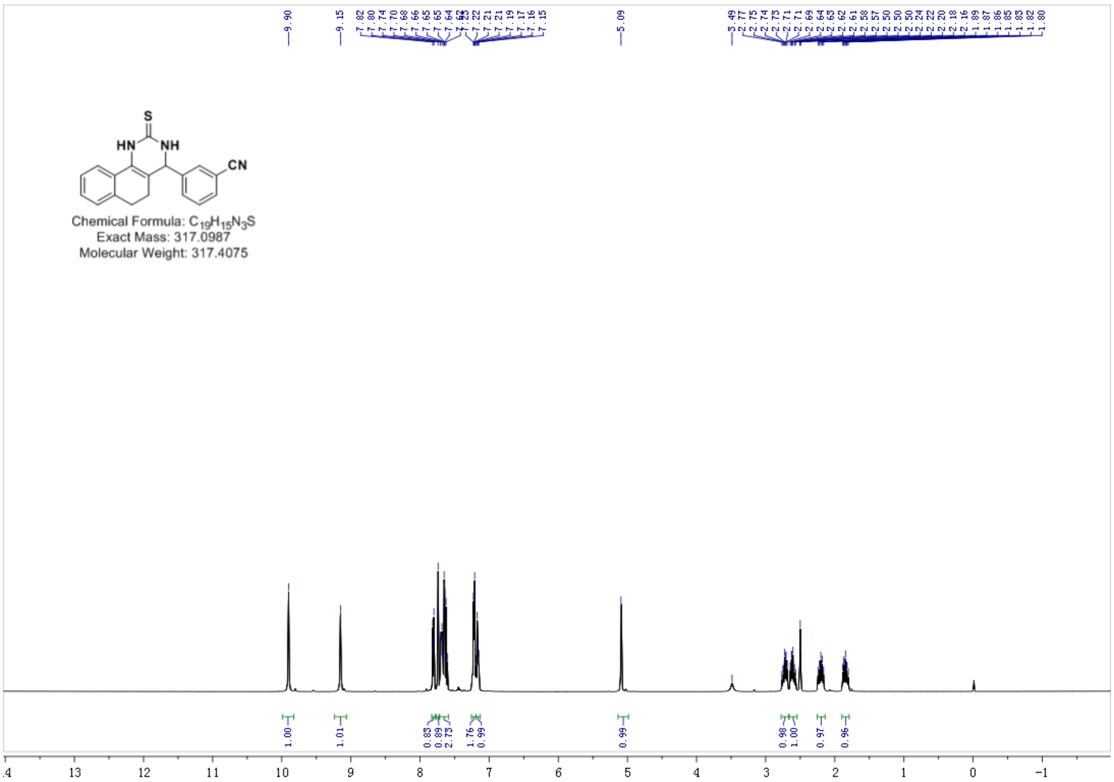

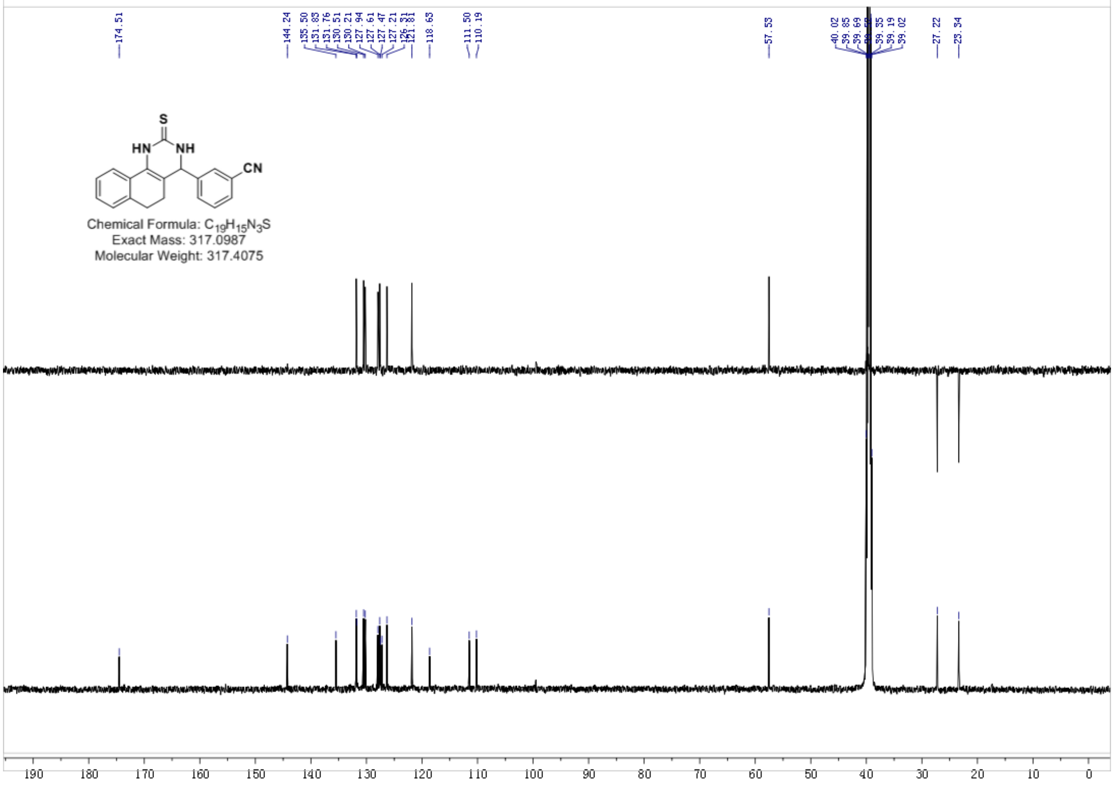

methyl 4-(2-thioxo-1,2,3,4,5,6-hexahydrobenzo[*h*]quinazolin-4-yl)benzoate (4f)

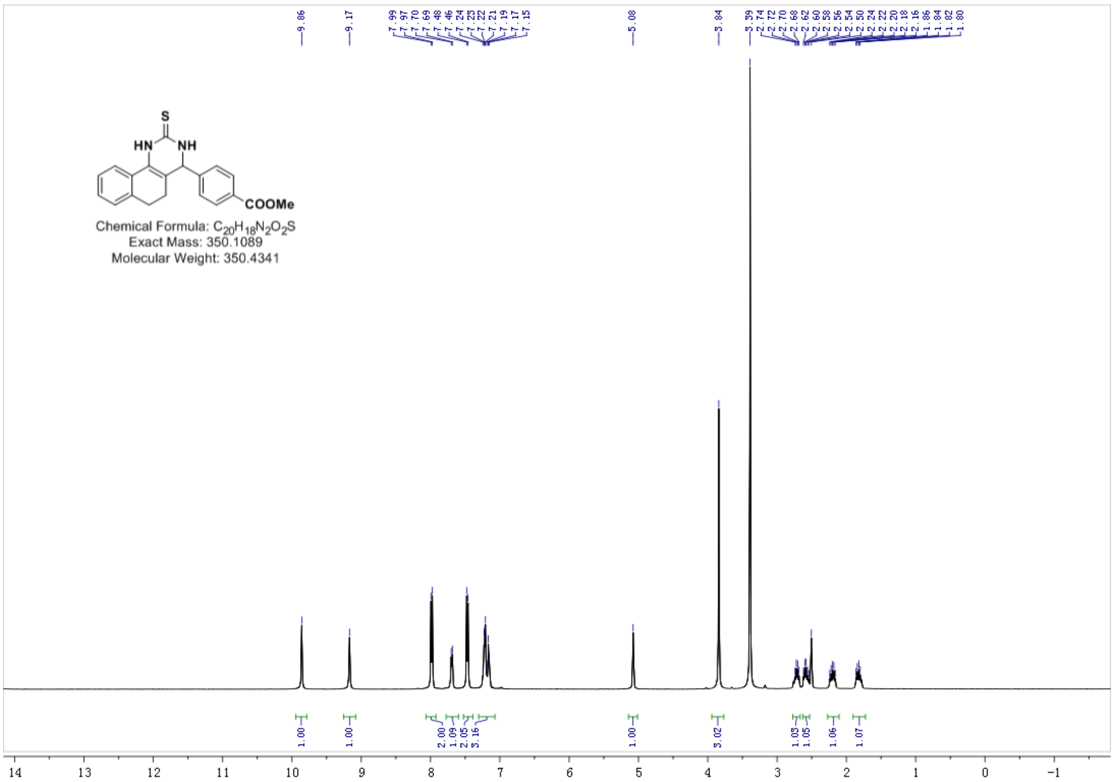

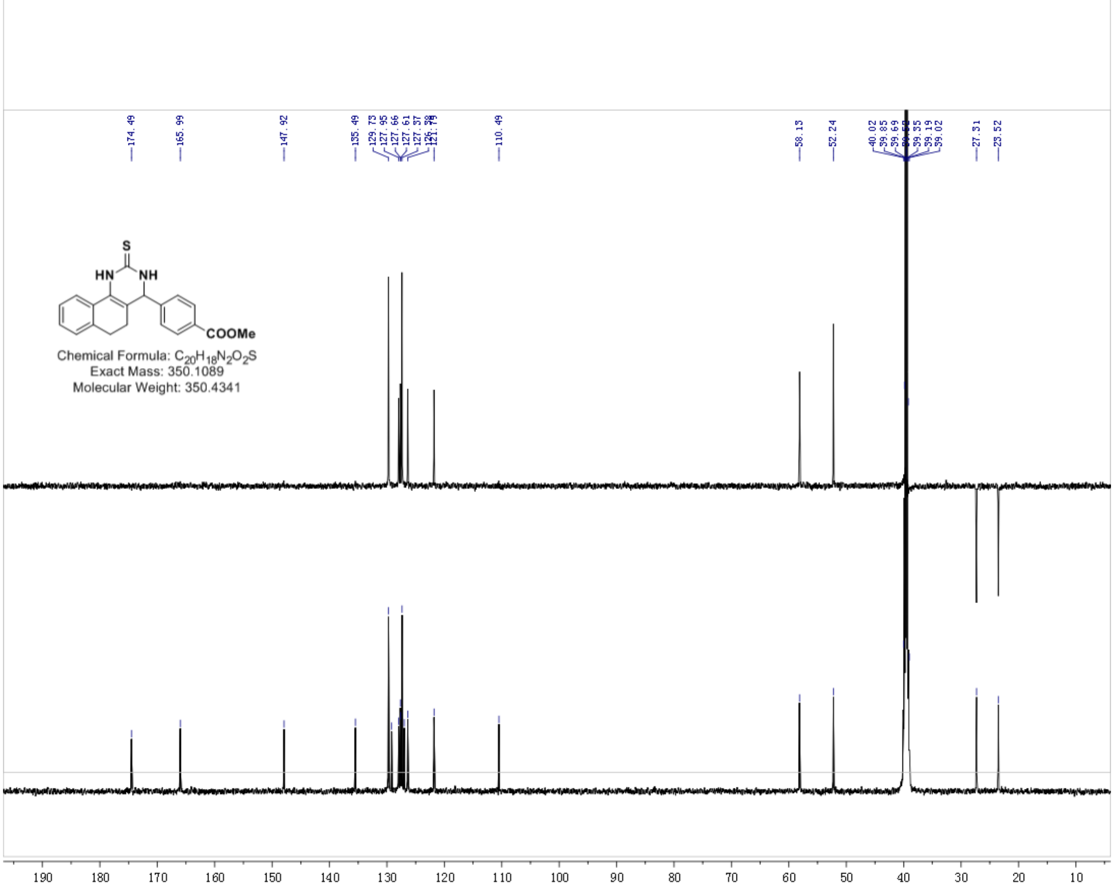

4-(4-fluorophenyl)-3,4,5,6-tetrahydrobenzo[*h*]quinazoline-2(*1H*)-thione (4g)

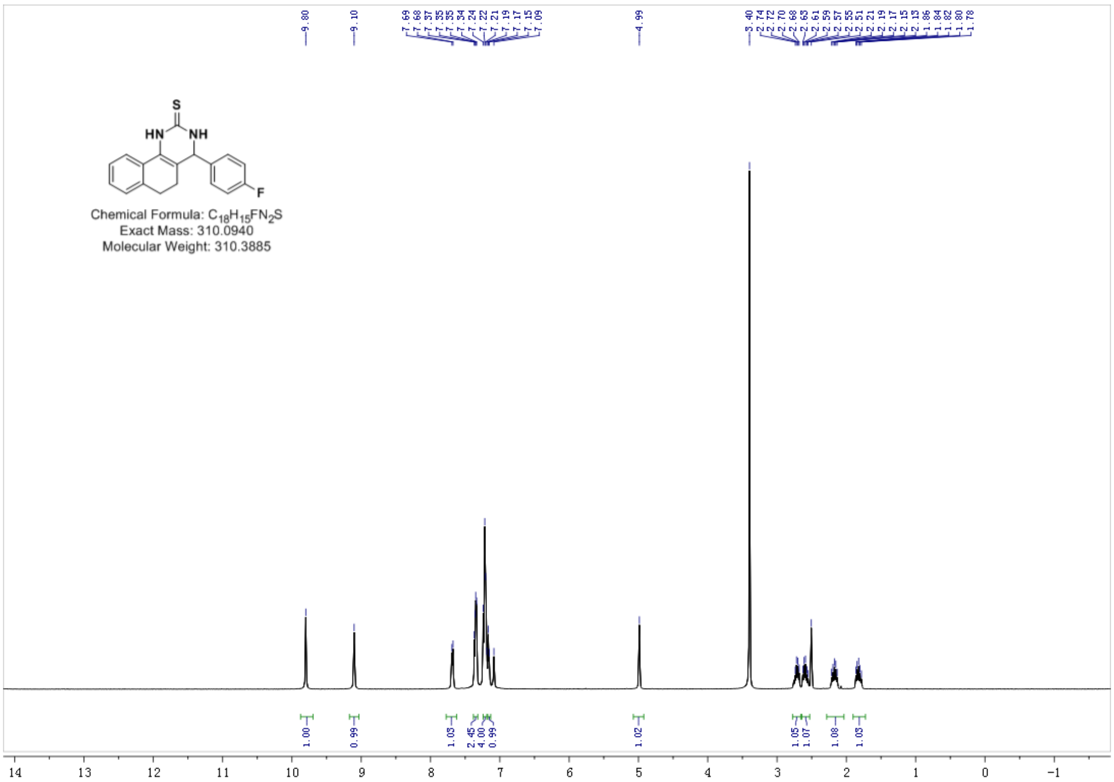

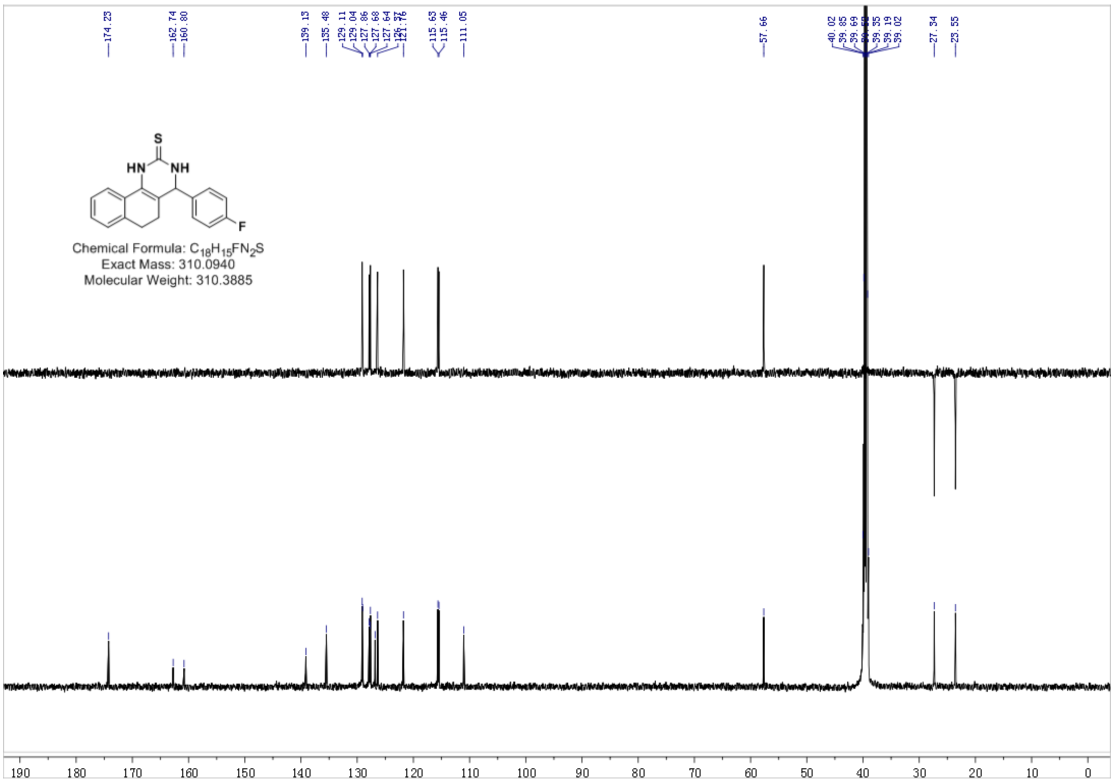

4-(4-chlorophenyl)-3,4,5,6-tetrahydrobenzo[*h*]quinazoline-2(*1H*)-thione (4h)

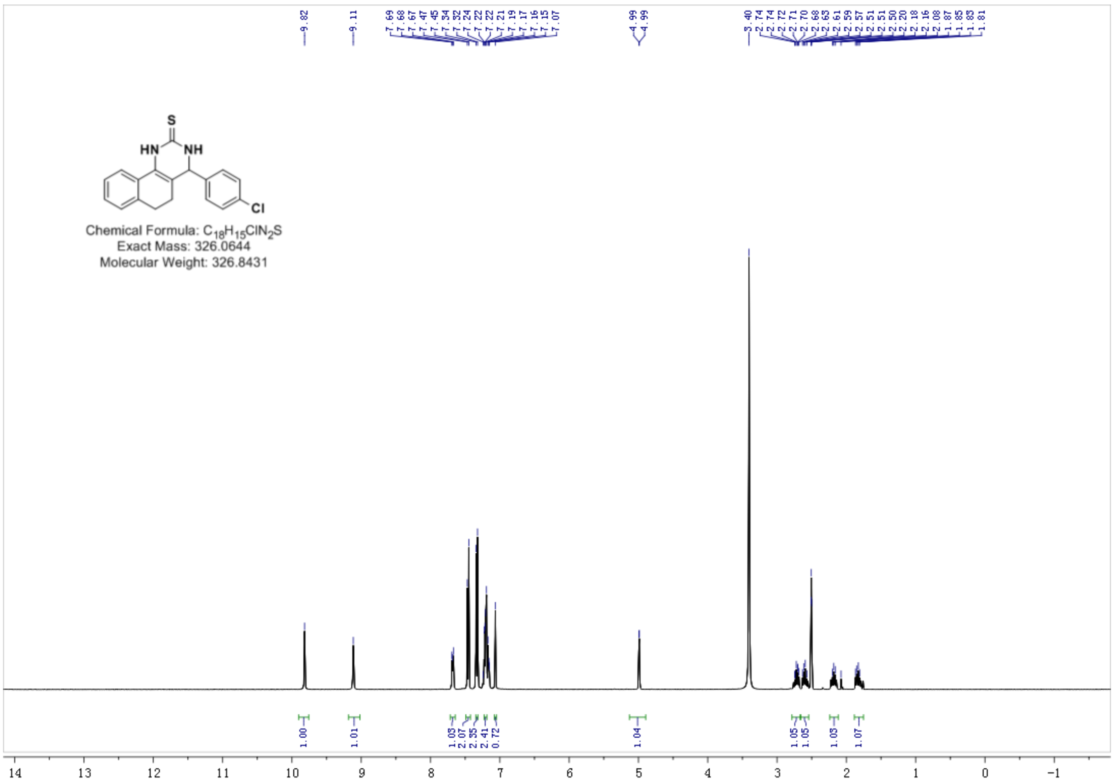

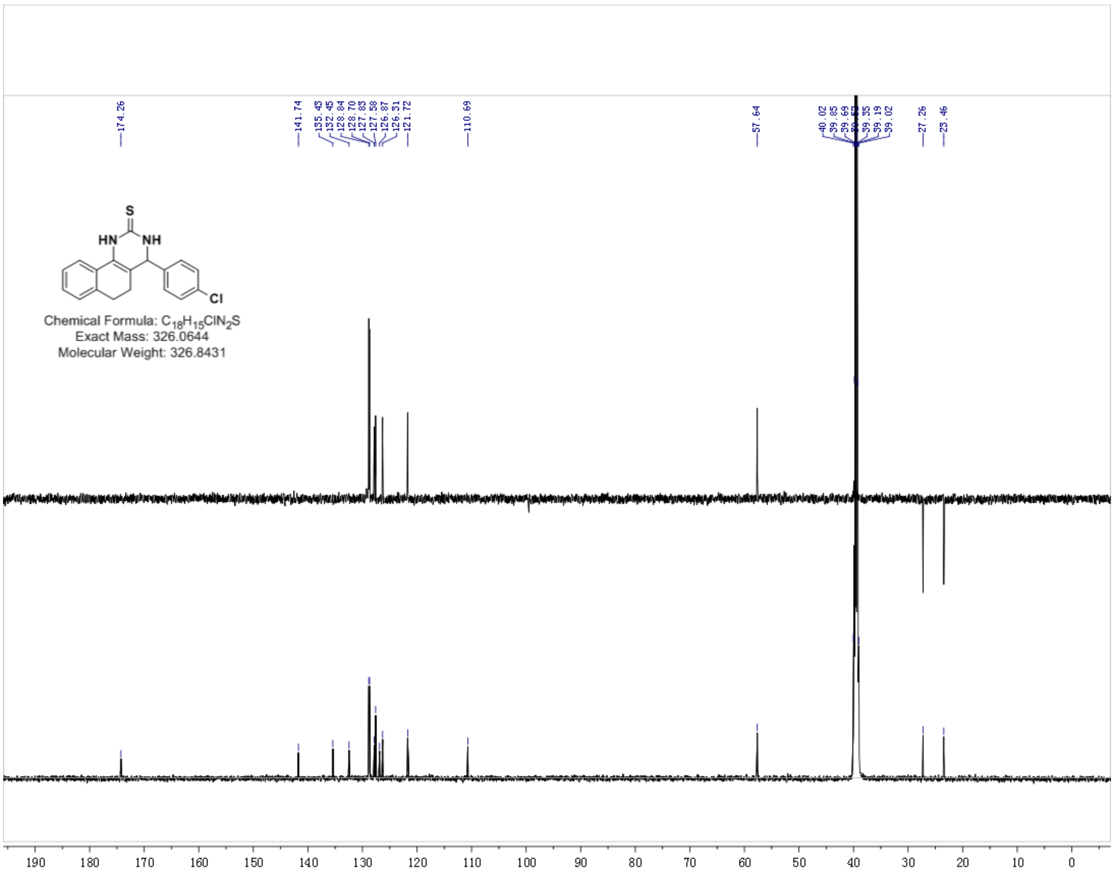

4-(4-bromophenyl)-3,4,5,6-tetrahydrobenzo[*h*]quinazoline-2(*1H*)-thione (4i)

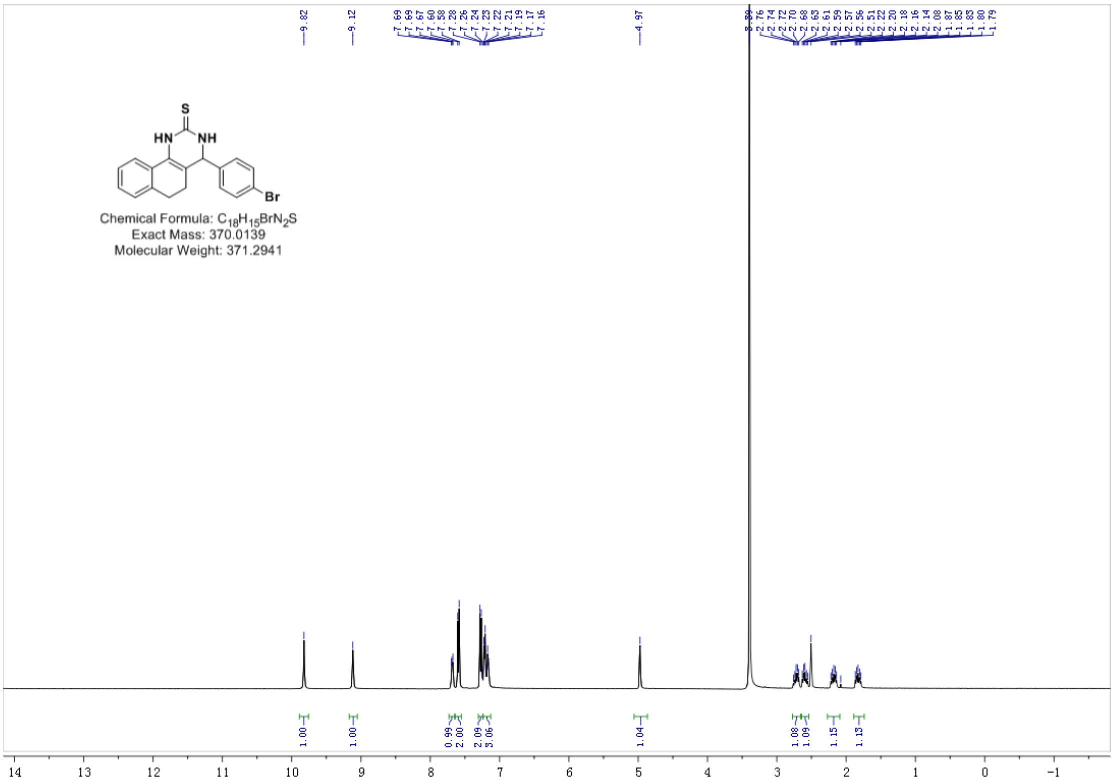

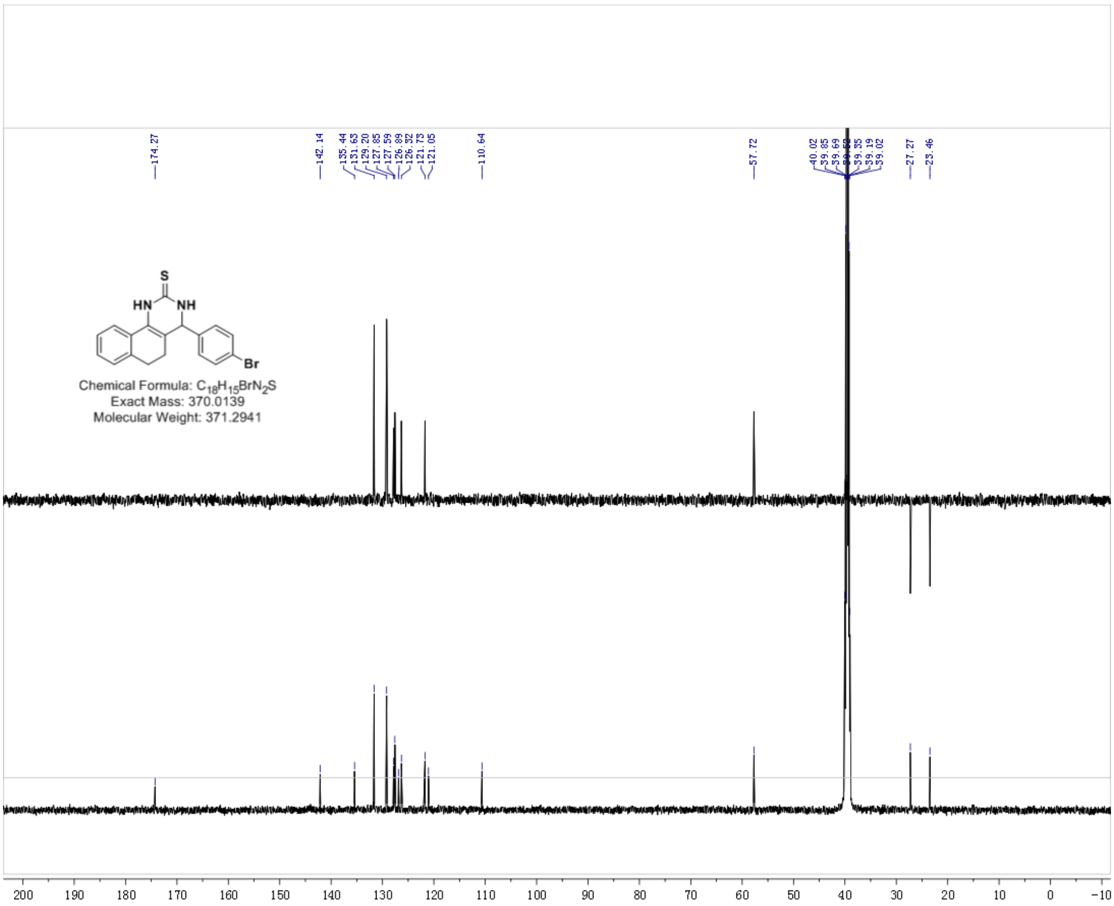

8-methoxy-4-phenyl-3,4,5,6-tetrahydrobenzo[*h*]quinazoline-2(*1H*)-thione (4j)

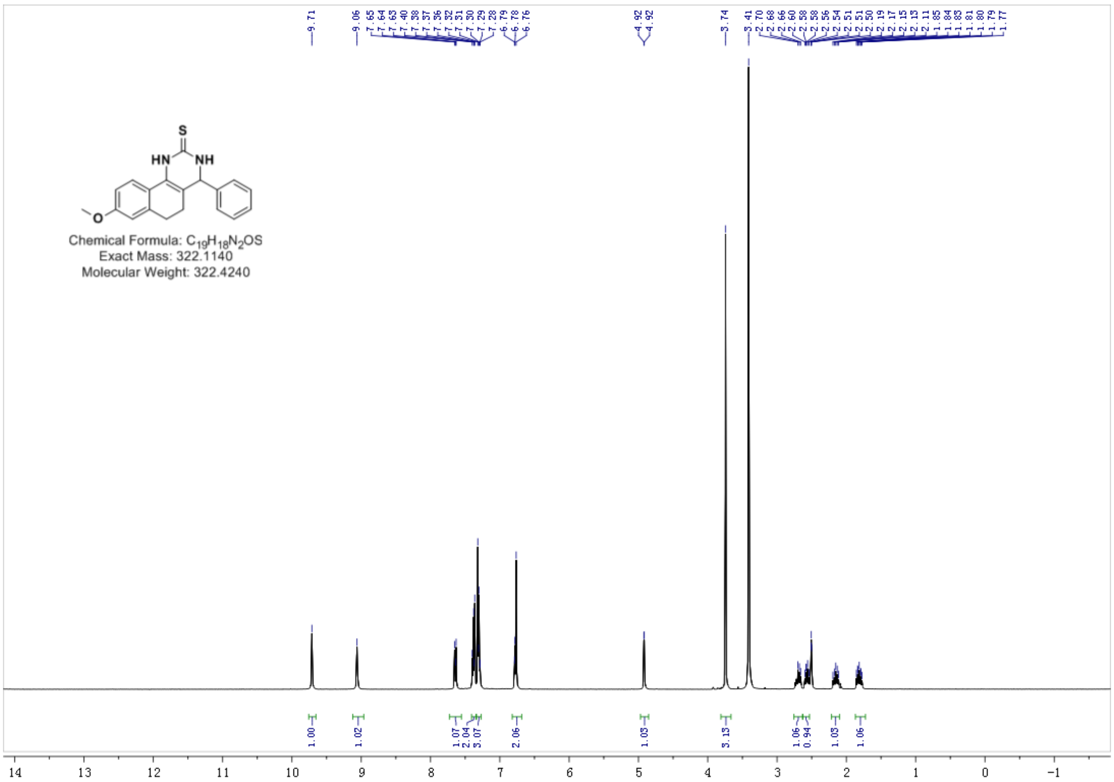

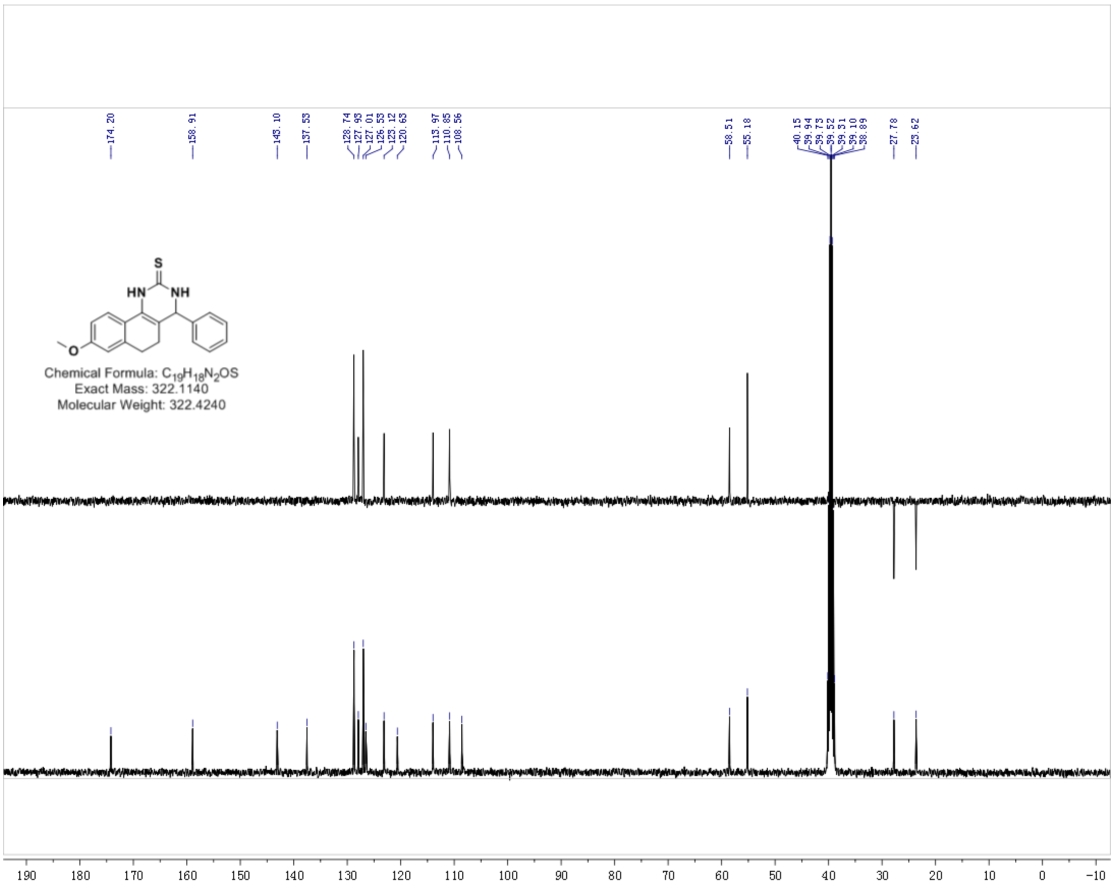

9-bromo-4-phenyl-3,4,5,6-tetrahydrobenzo[*h*]quinazoline-2(*1H*)-thione (4k)

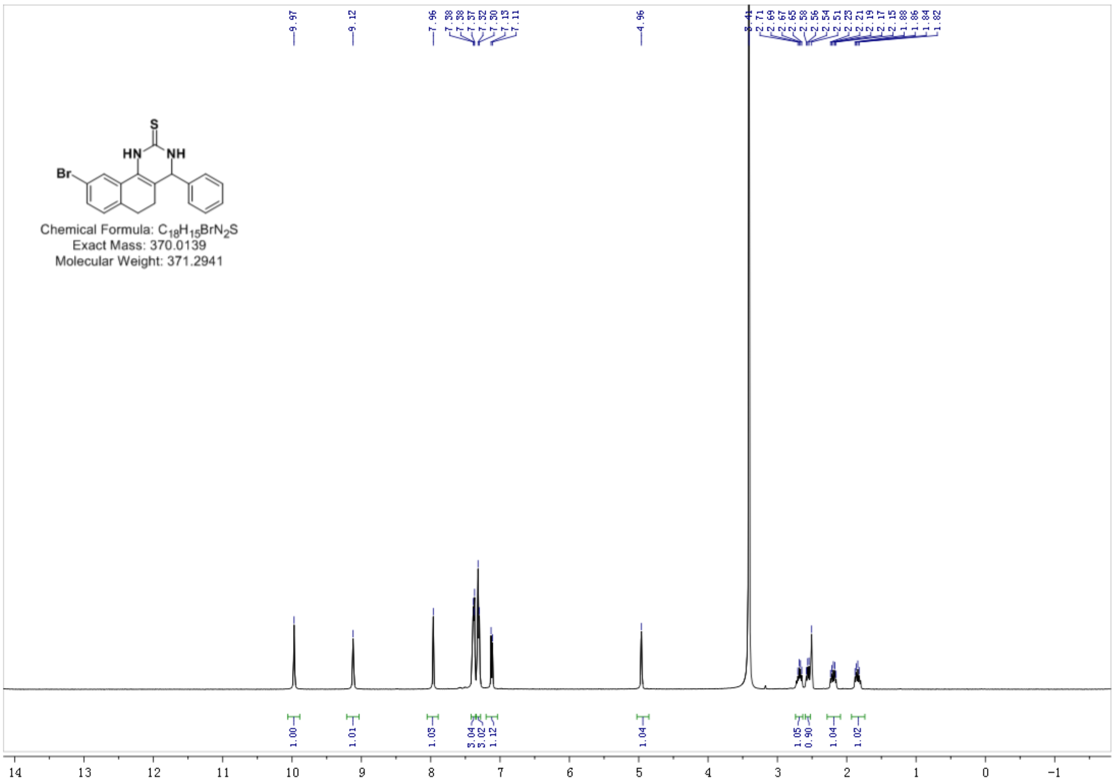

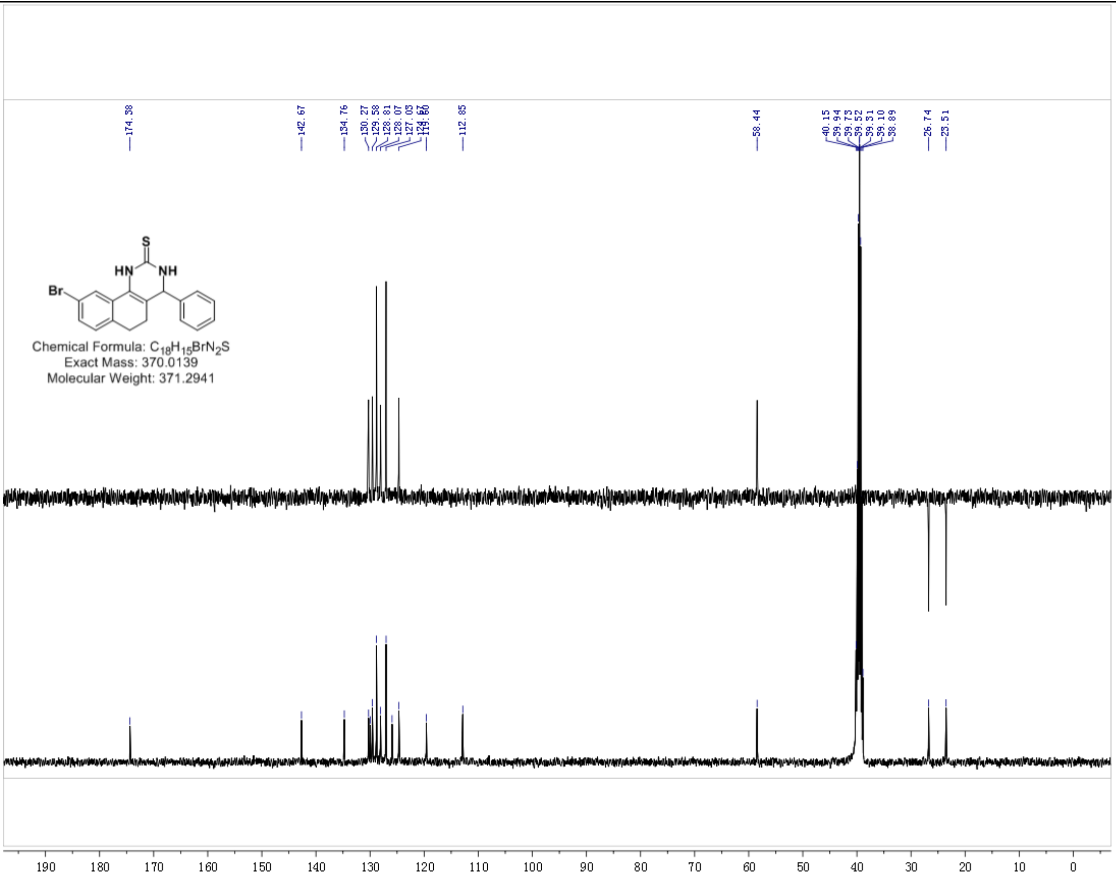

9-nitro-4-phenyl-3,4,5,6-tetrahydrobenzo[*h*]quinazoline-2(*1H*)-thione (4l)

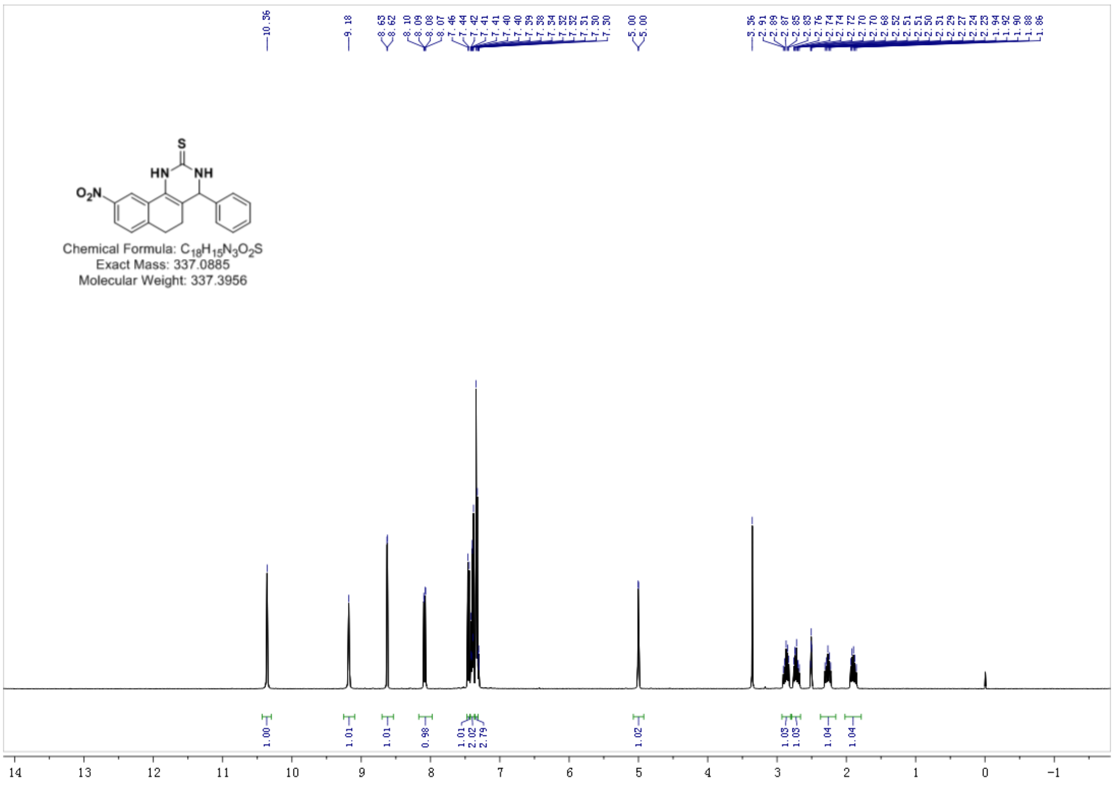

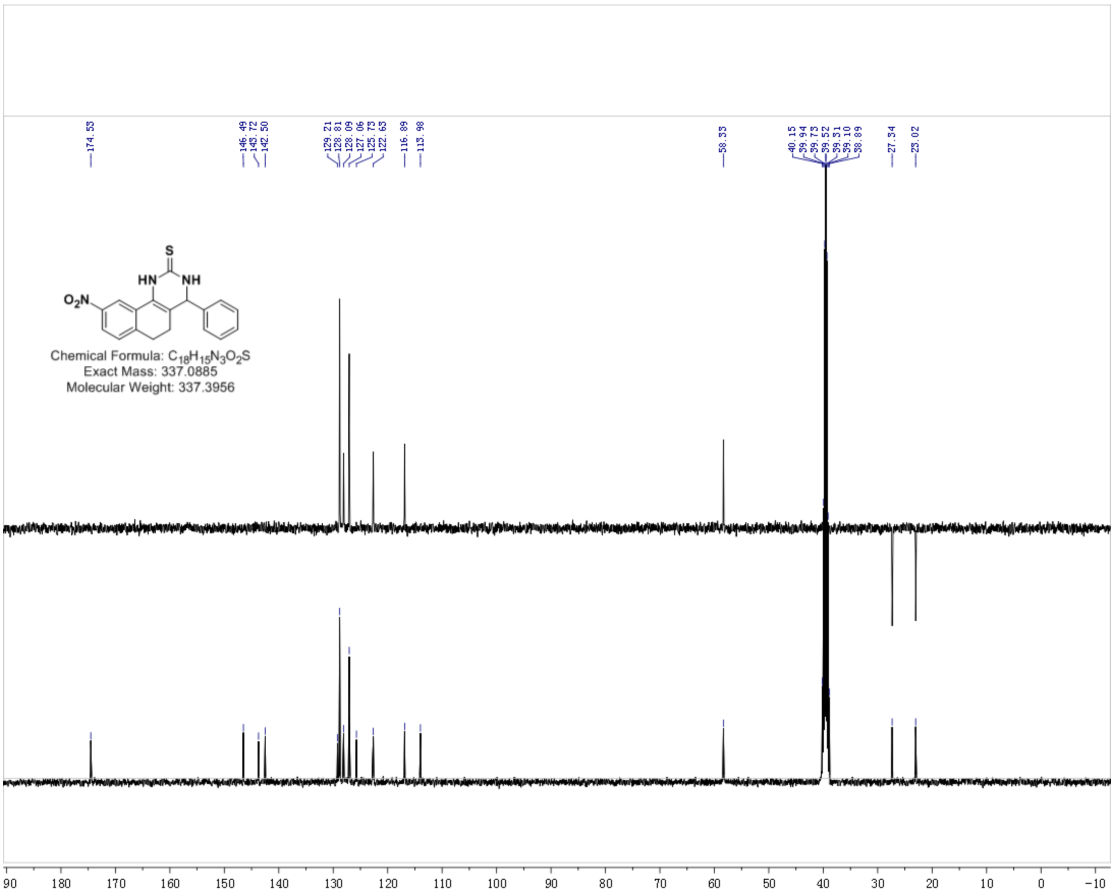

4-phenyl-3,4-dihydro-*1H*-indeno[1,2-*d*]pyrimidine-2(*5H*)-thione (4m)

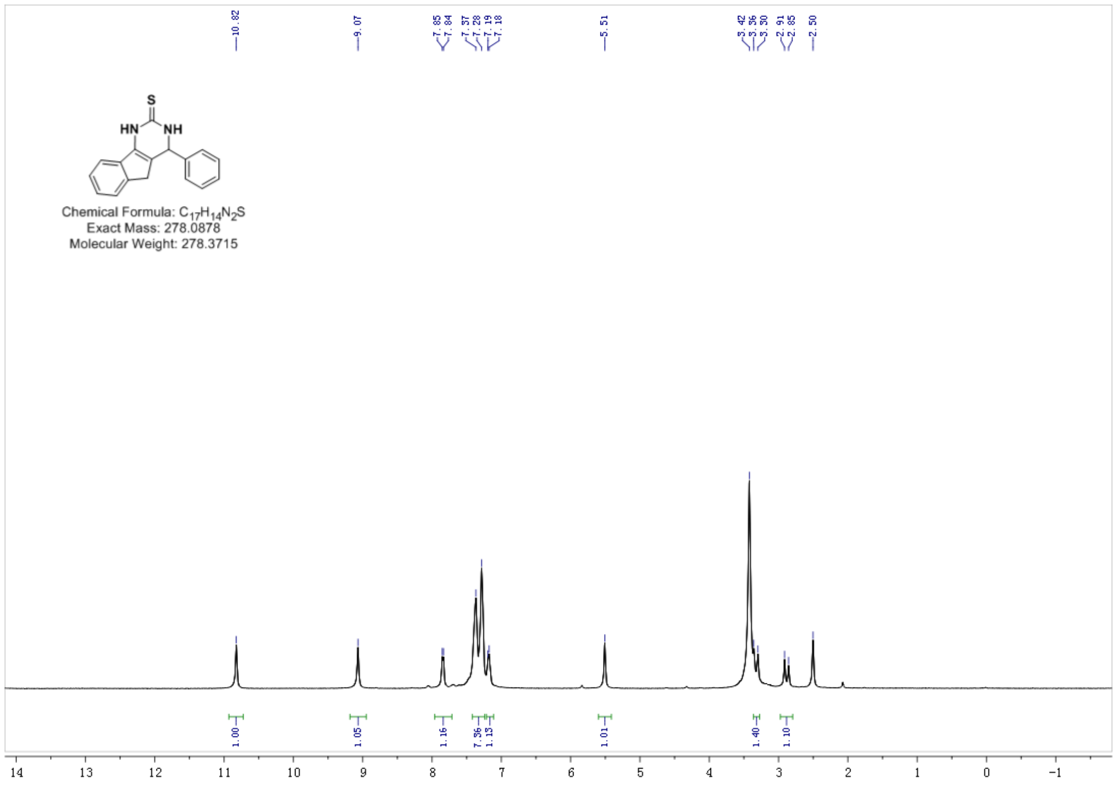

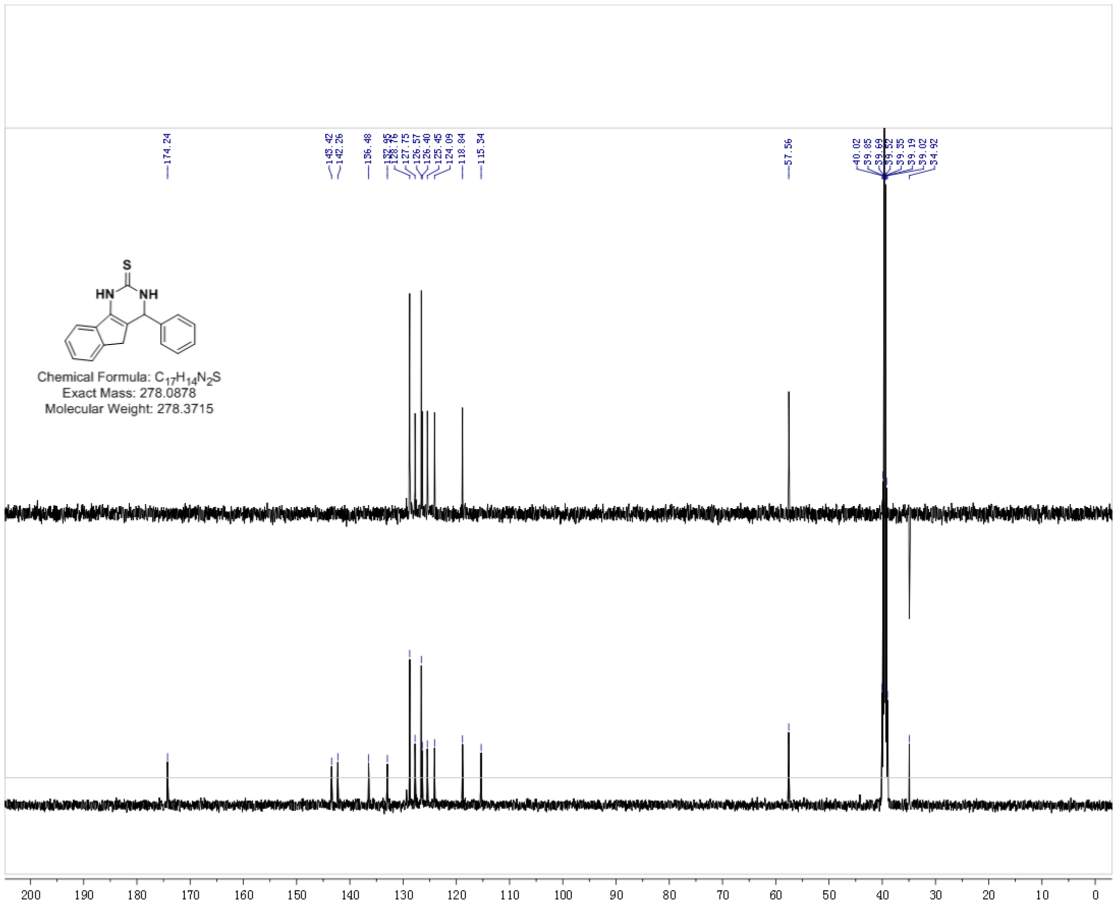

4-phenyl-3,4,5,6-tetrahydrobenzo[*h*]quinazolin-2(*1H*)-one (6a)

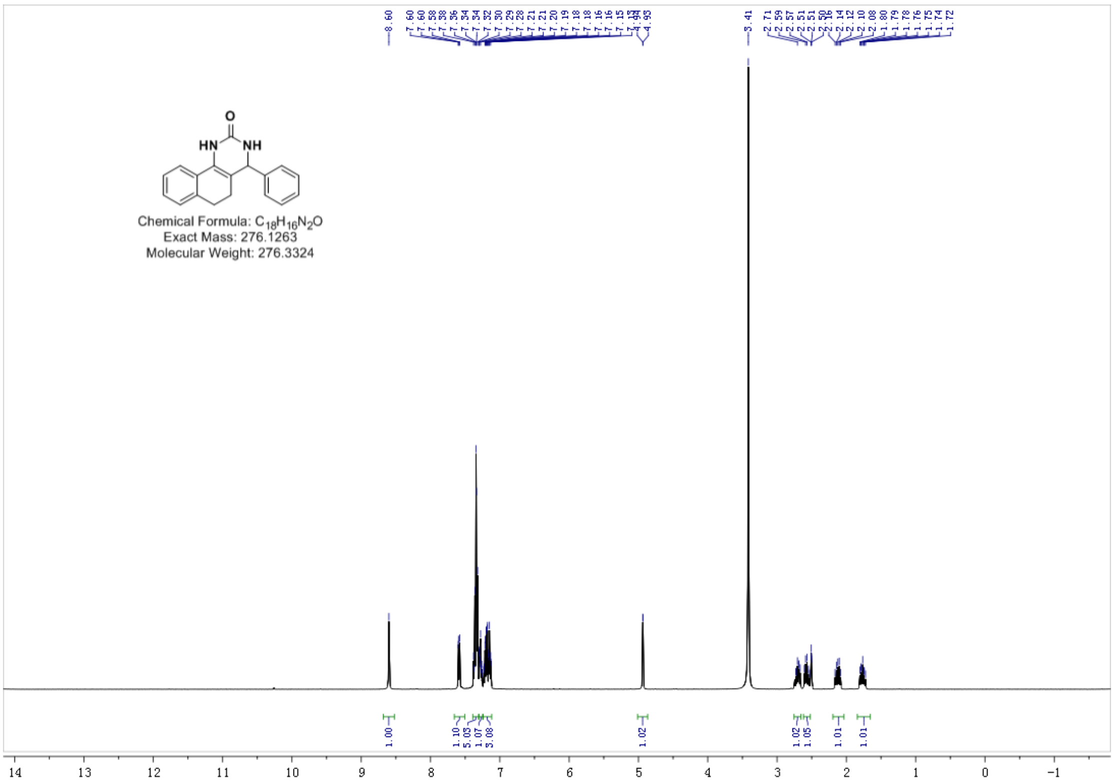

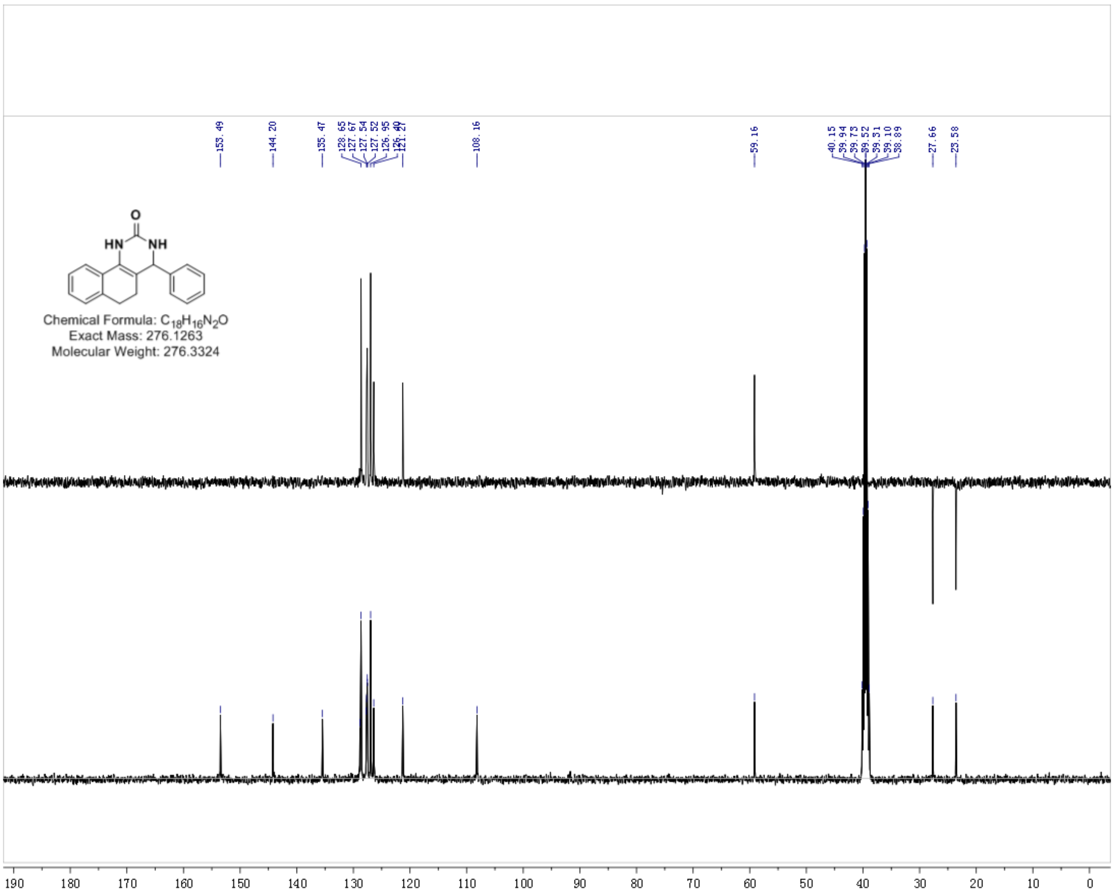

4-*m*-tolyl-3,4,5,6-tetrahydrobenzo[*h*]quinazolin-2(*1H*)-one (6b)

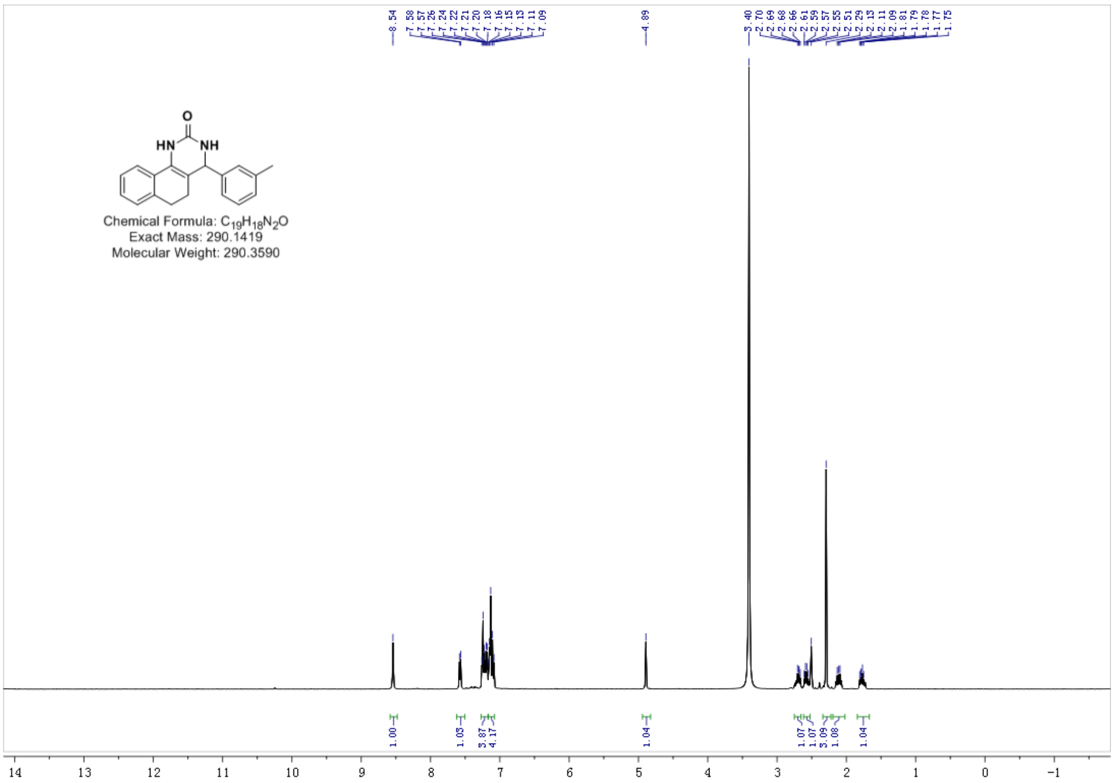

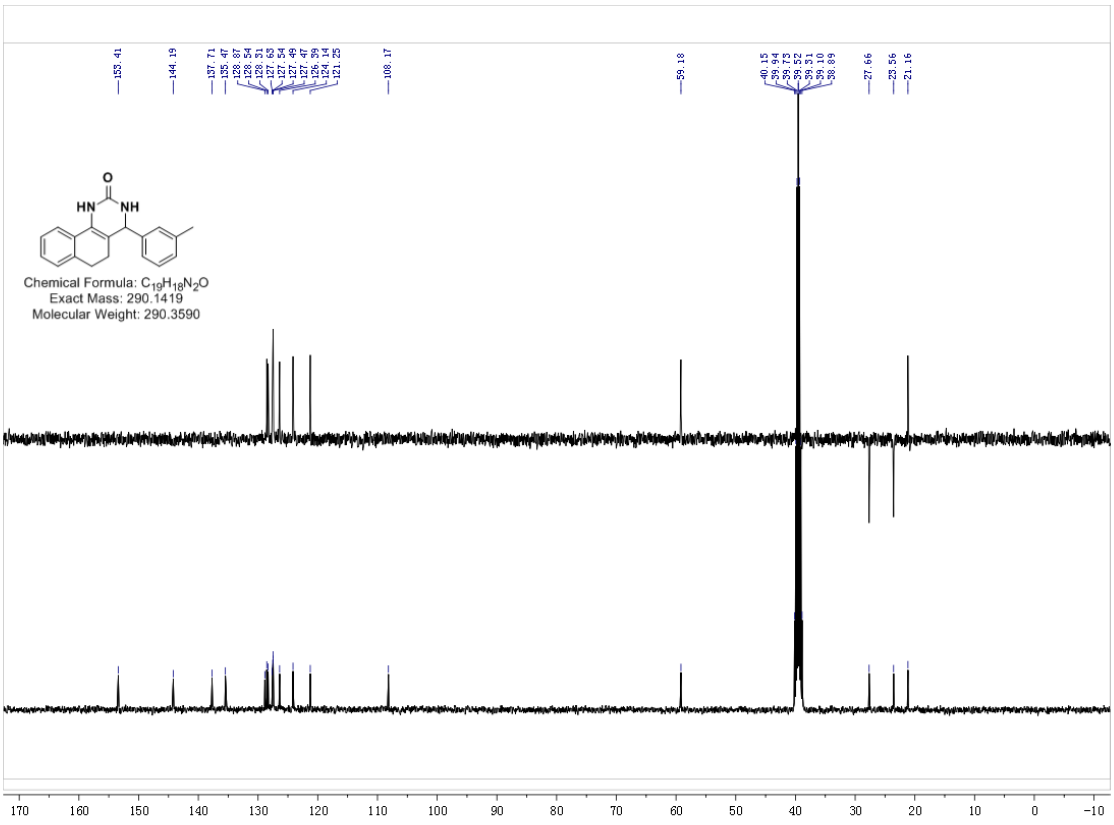

4-(4-nitrophenyl)-3,4,5,6-tetrahydrobenzo[*h*]quinazolin-2(*1H*)-one (6c)

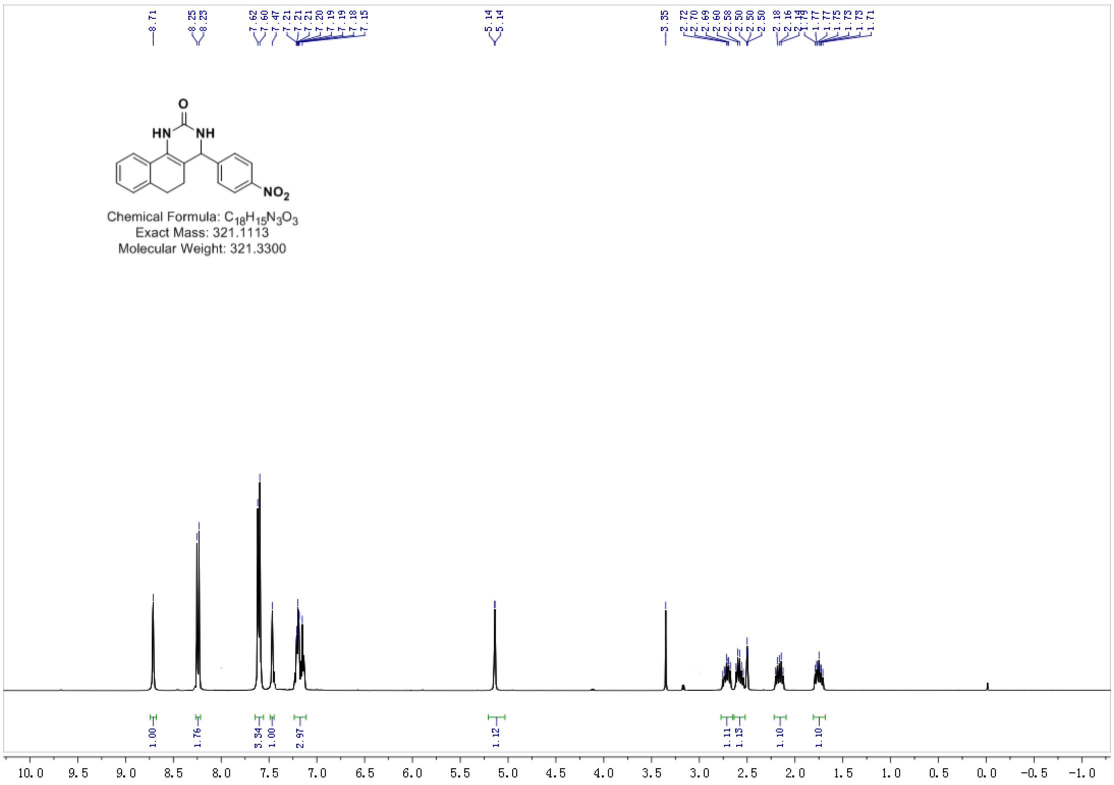

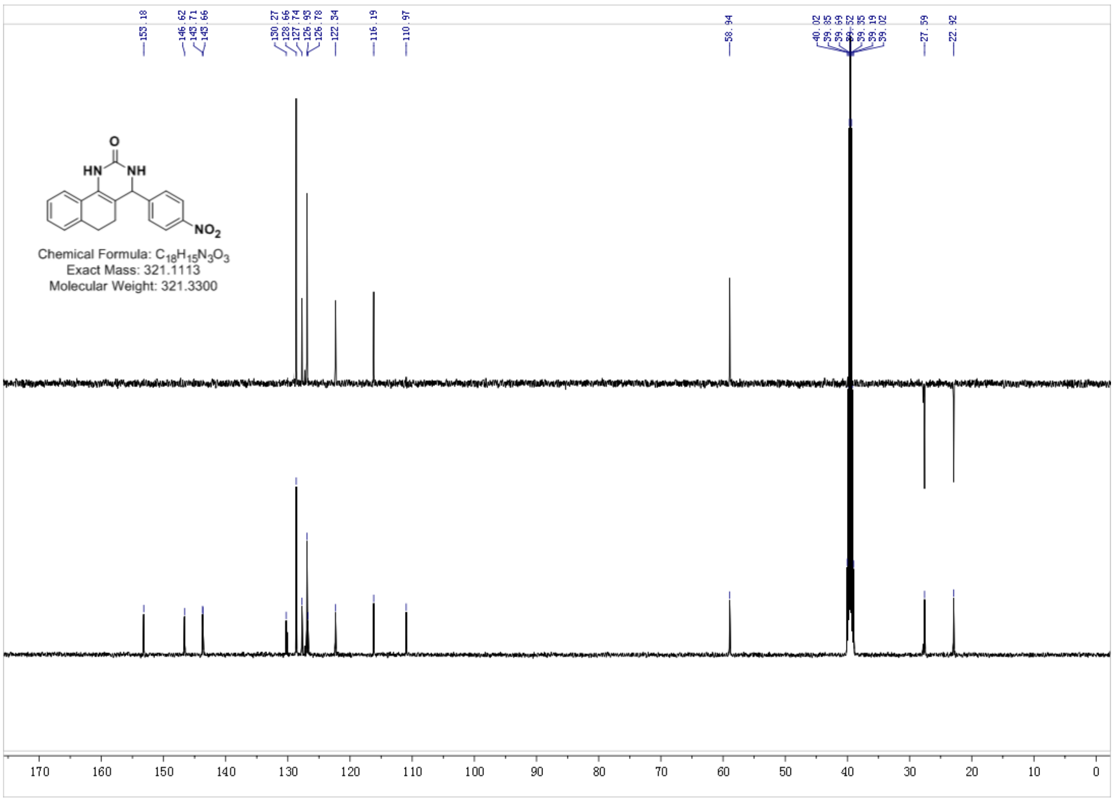

3-(2-oxo-1,2,3,4,5,6-hexahydrobenzo[*h*]quinazolin-4-yl)benzonitrile (6d)

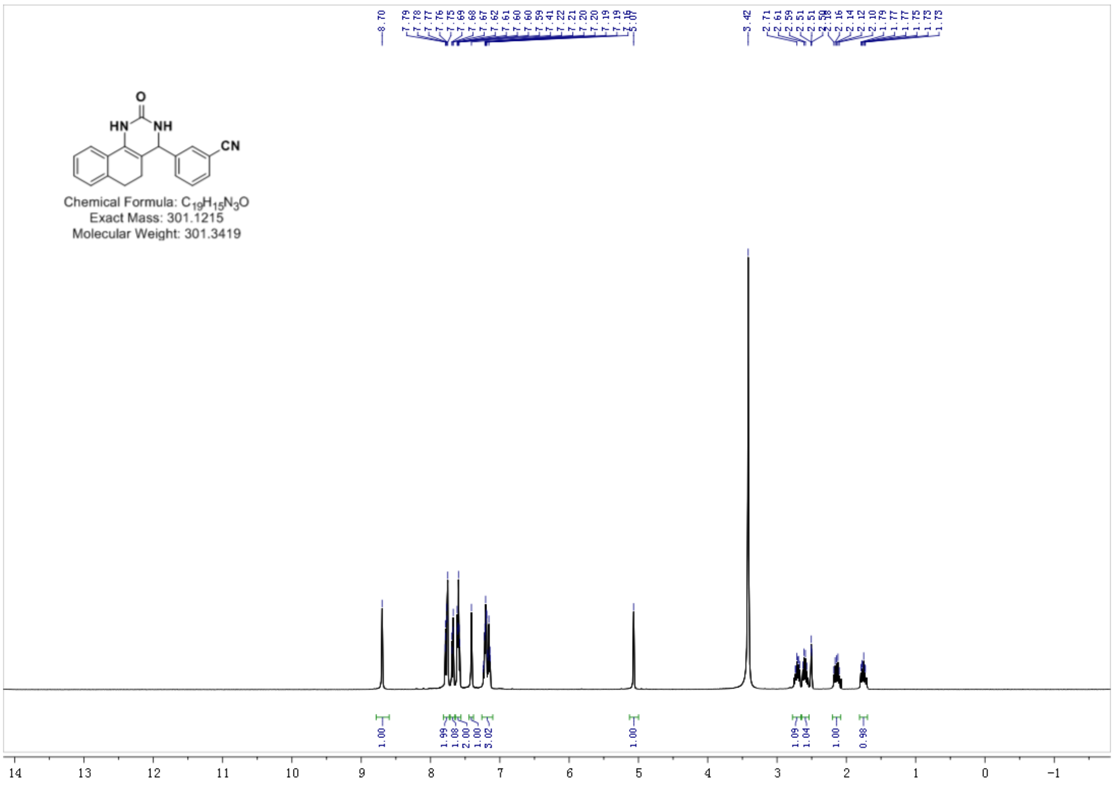

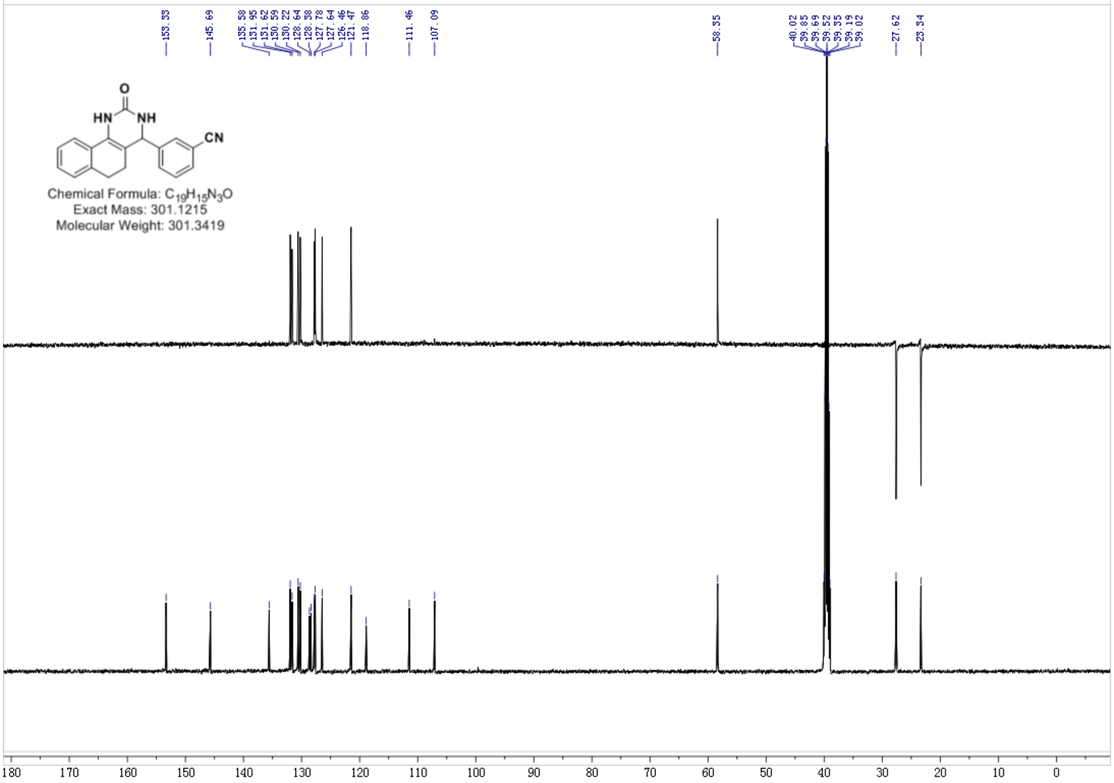

4-(4-fluorophenyl)-3,4,5,6-tetrahydrobenzo[*h*]quinazolin-2(*1H*)-one (6e)

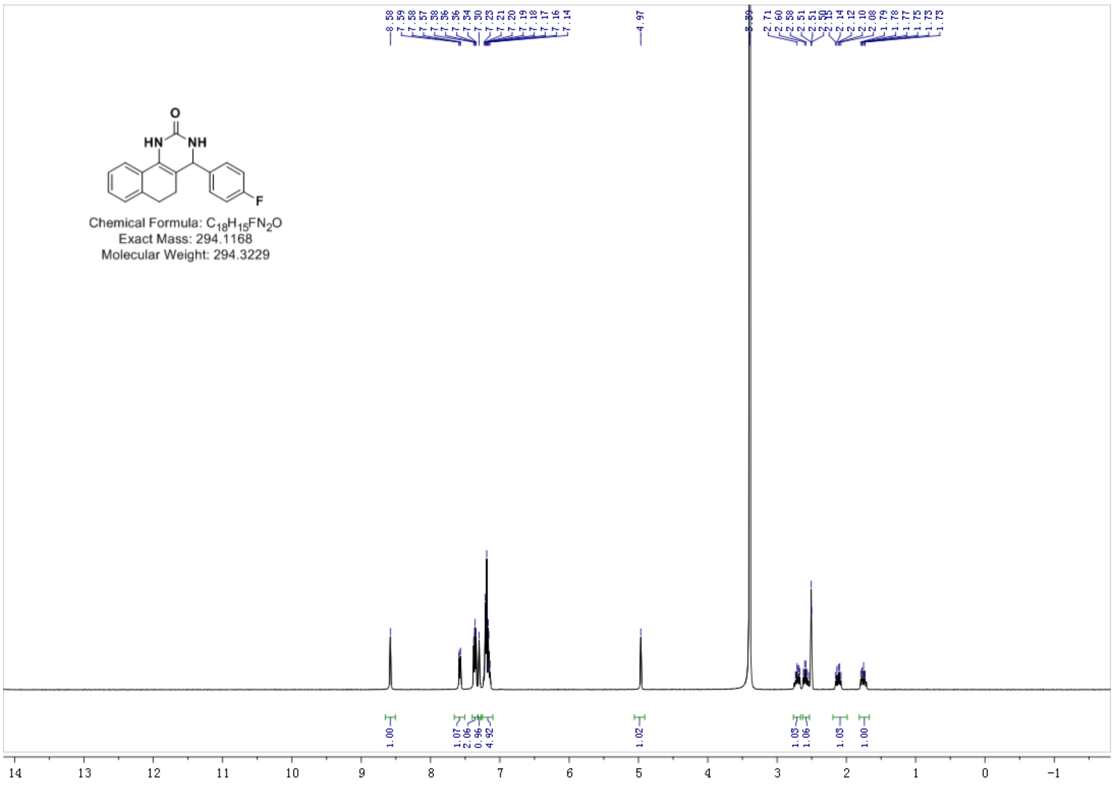

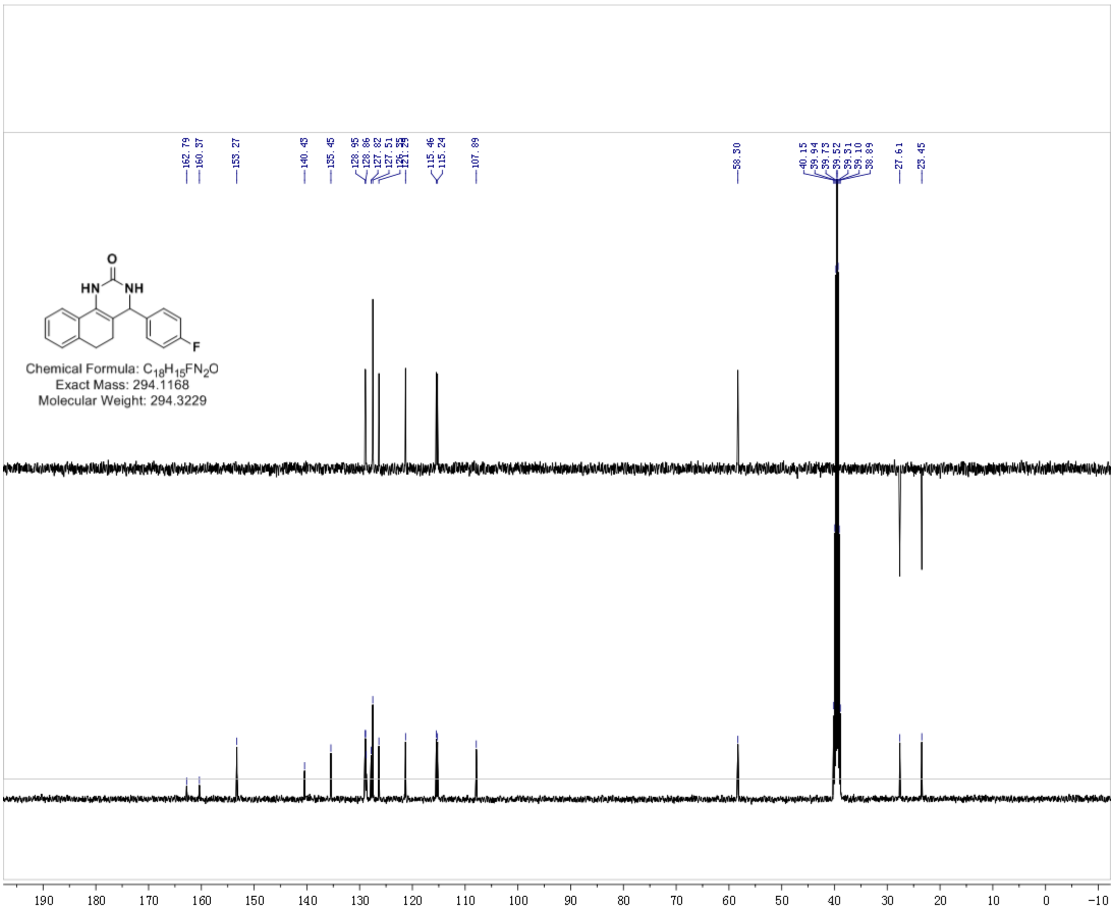

4-(3-chlorophenyl)-3,4,5,6-tetrahydrobenzo[*h*]quinazolin-2(*1H*)-one (6f)

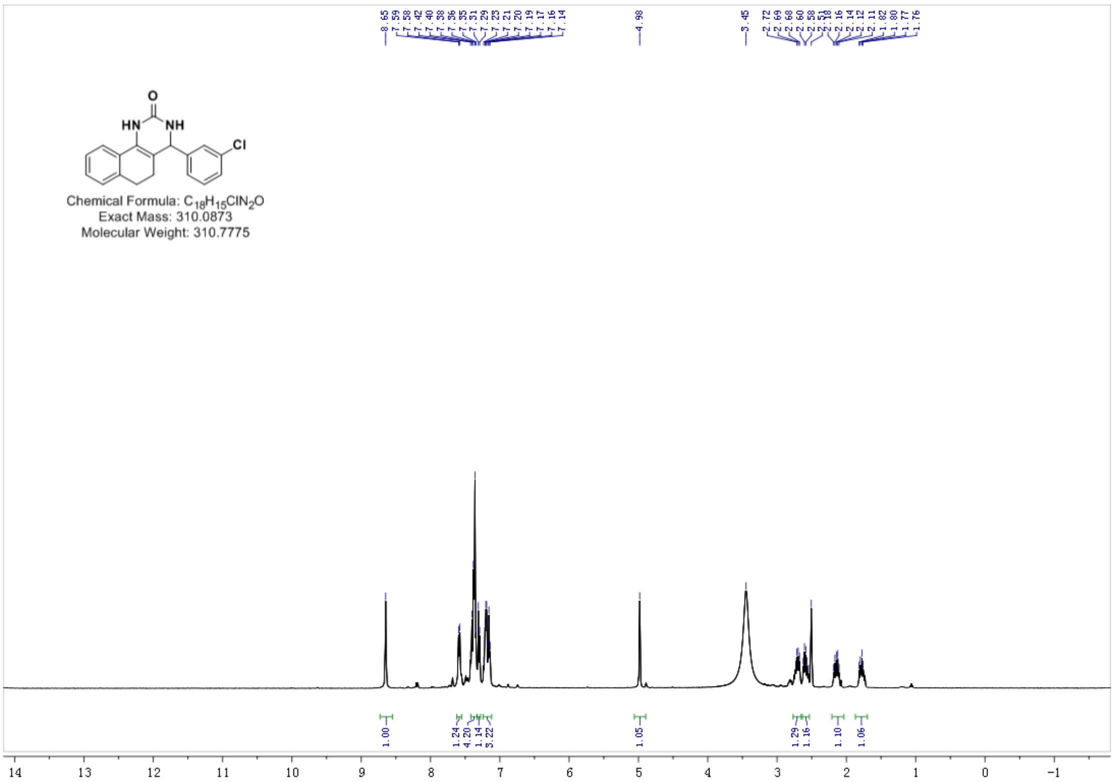

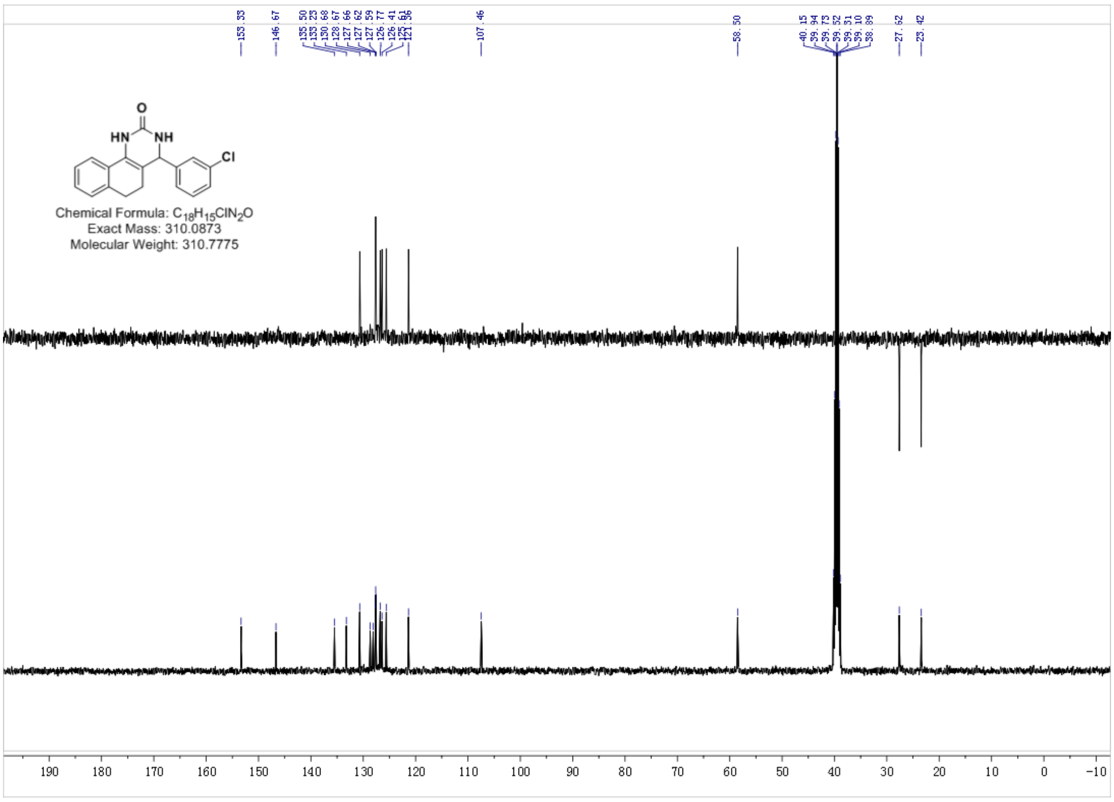

4-(2-bromophenyl)-3,4,5,6-tetrahydrobenzo[*h*]quinazolin-2(*1H*)-one (6g)

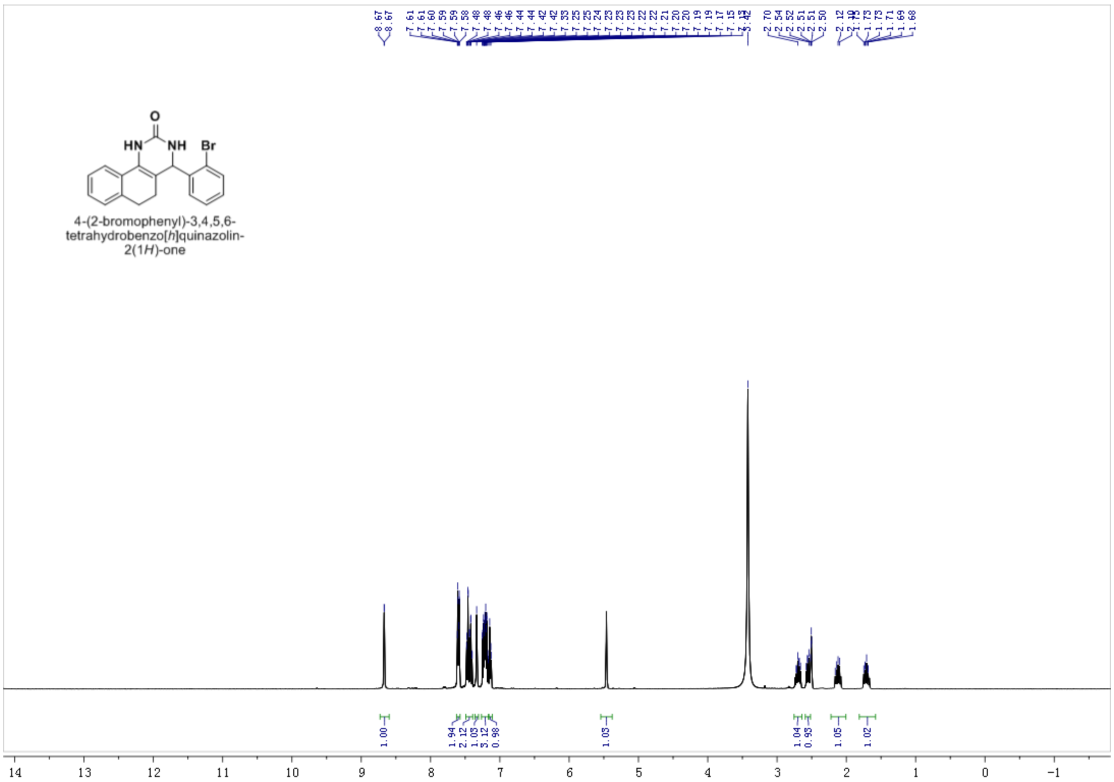

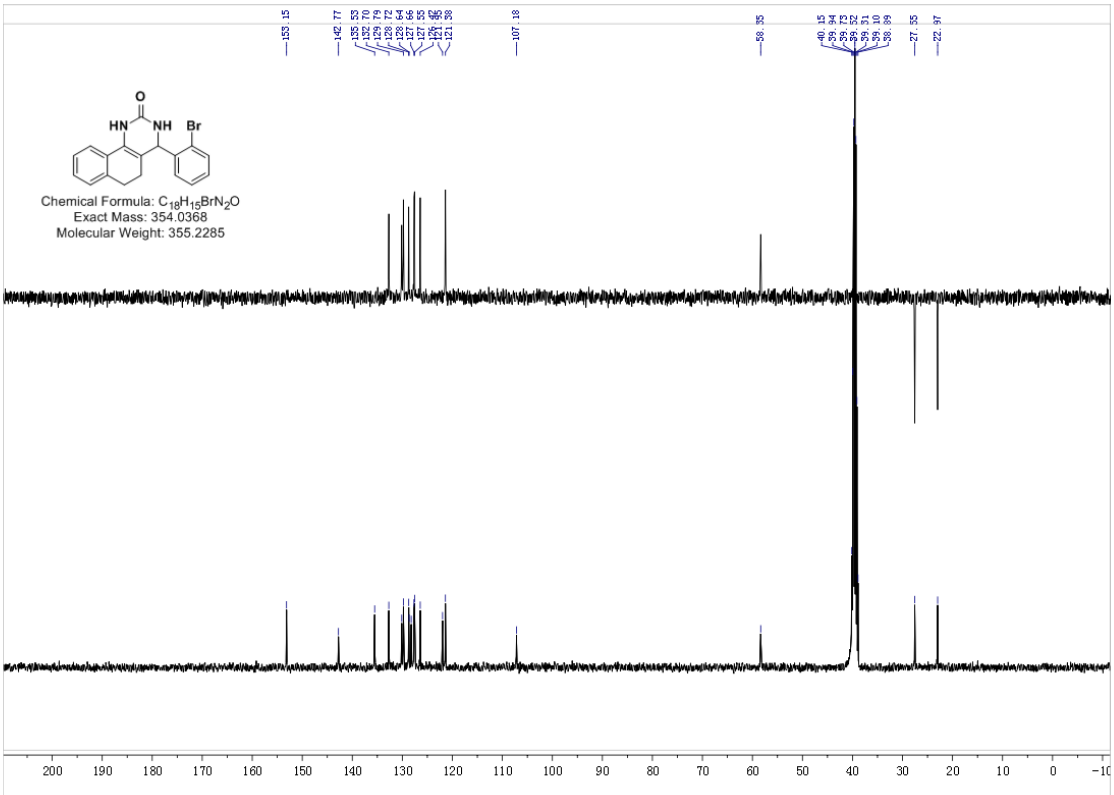

8-methoxy-4-phenyl-3,4,5,6-tetrahydrobenzo[*h*]quinazolin-2(*1H*)-one (6h)

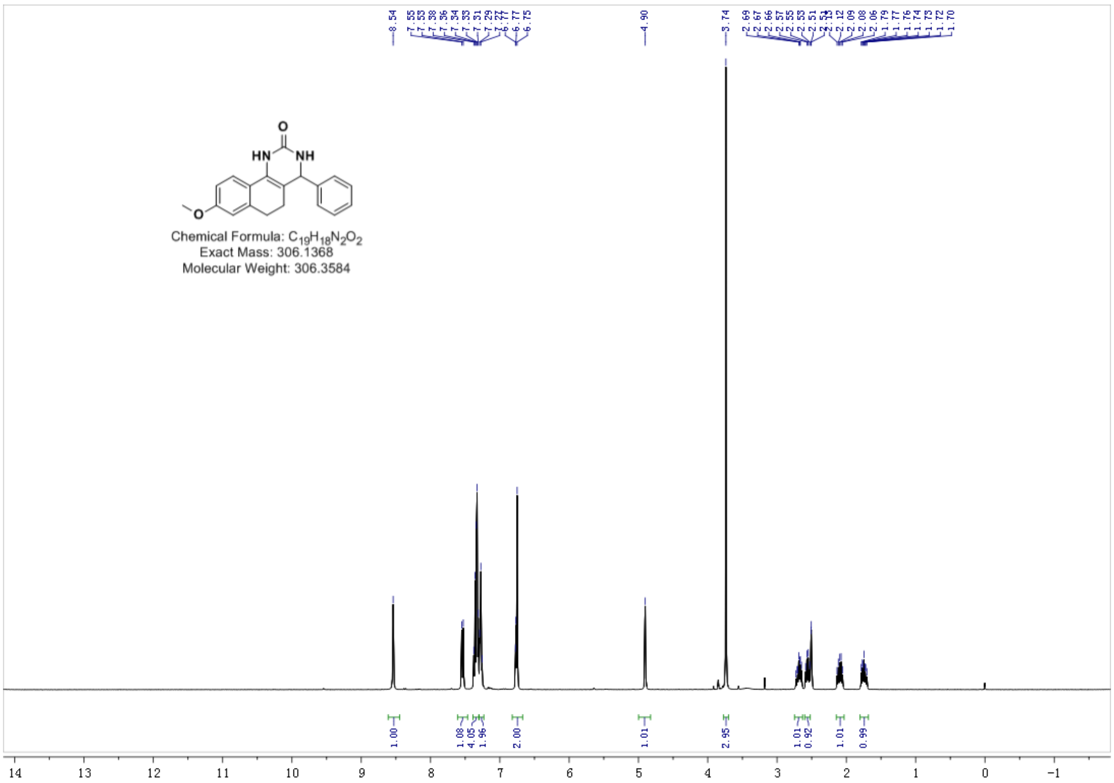

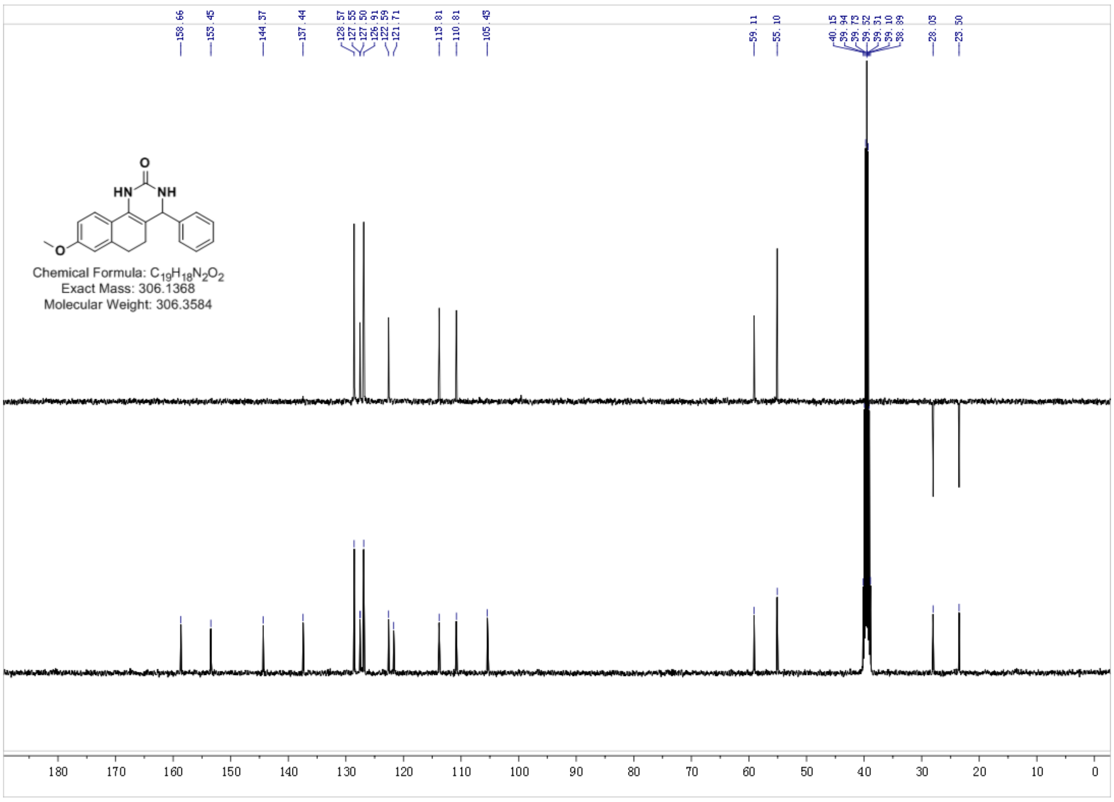

9-bromo-4-phenyl-3,4,5,6-tetrahydrobenzo[*h*]quinazolin-2(*1H*)-one (6i)

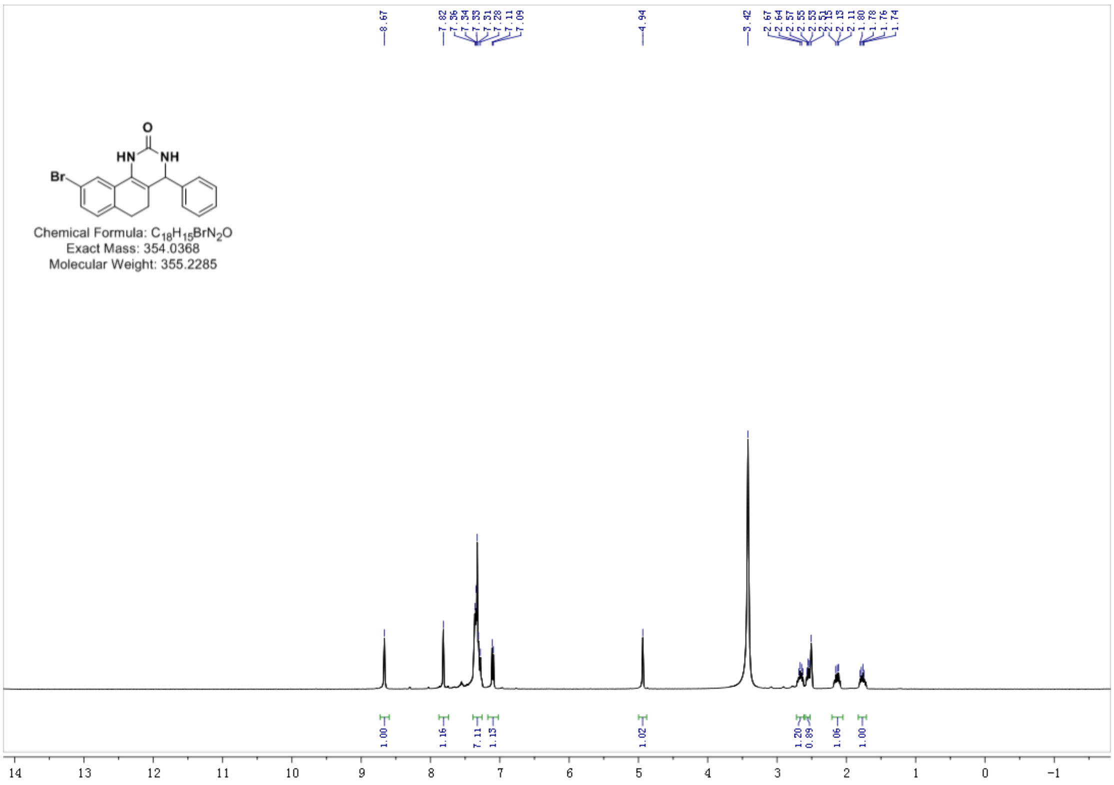

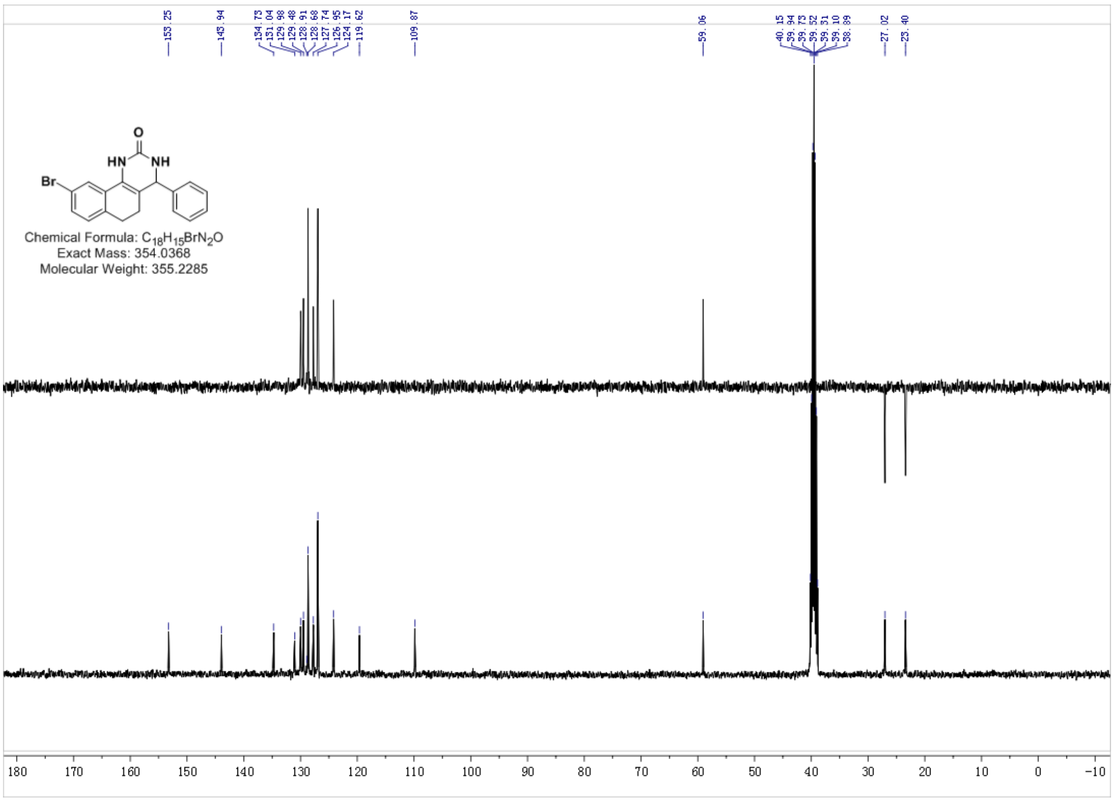

9-nitro-4-phenyl-3,4,5,6-tetrahydrobenzo[*h*]quinazolin-2(*1H*)-one (6j)

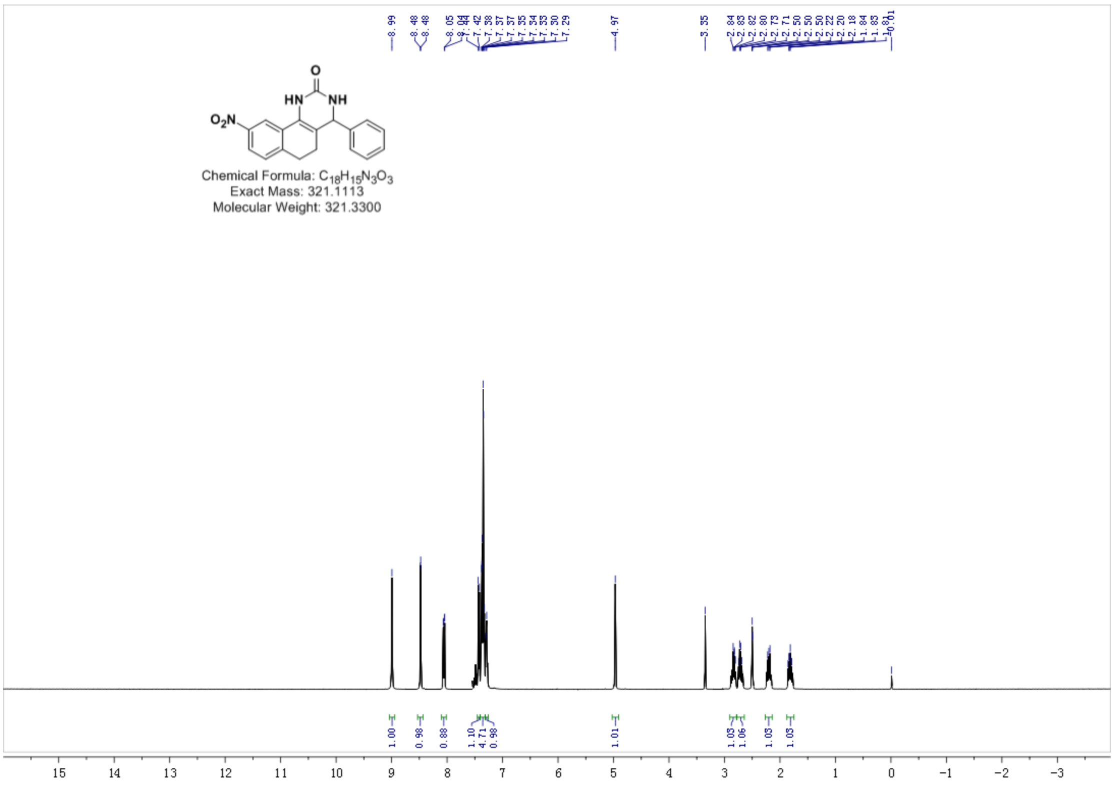

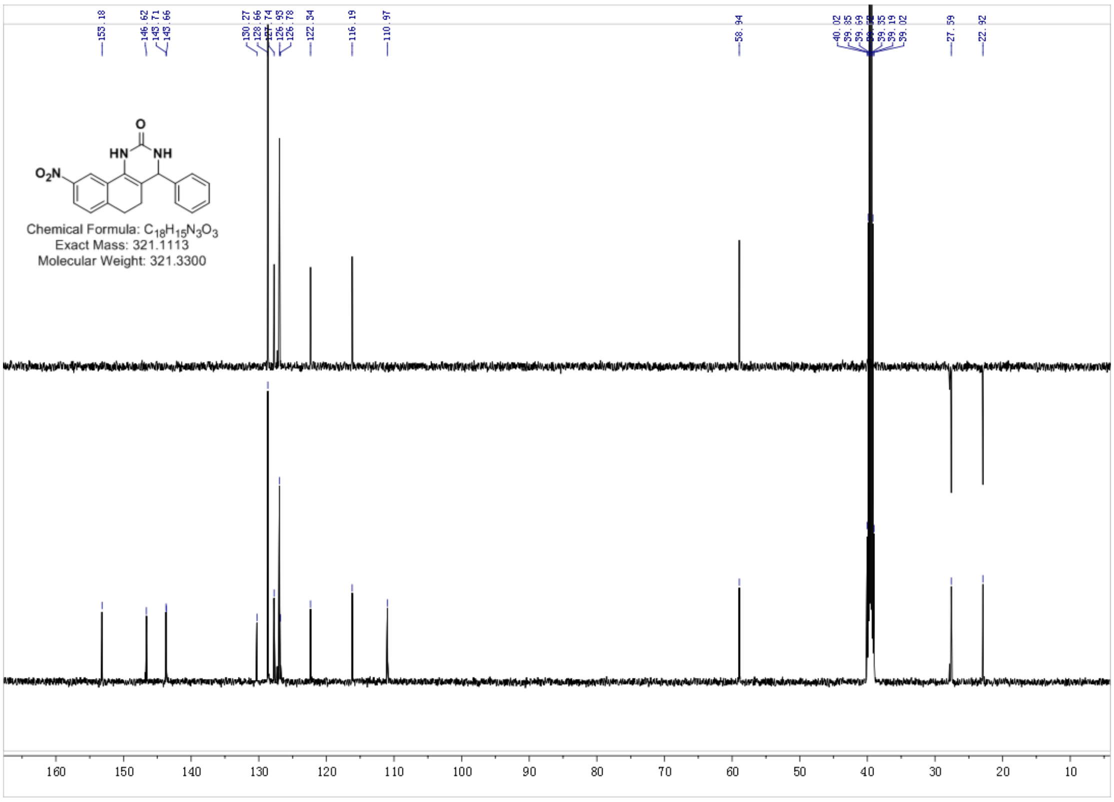

4-phenyl-3,4-dihydro-*1H*-indeno[1,2-*d*]pyrimidin-2(*5H*)-one (6k)

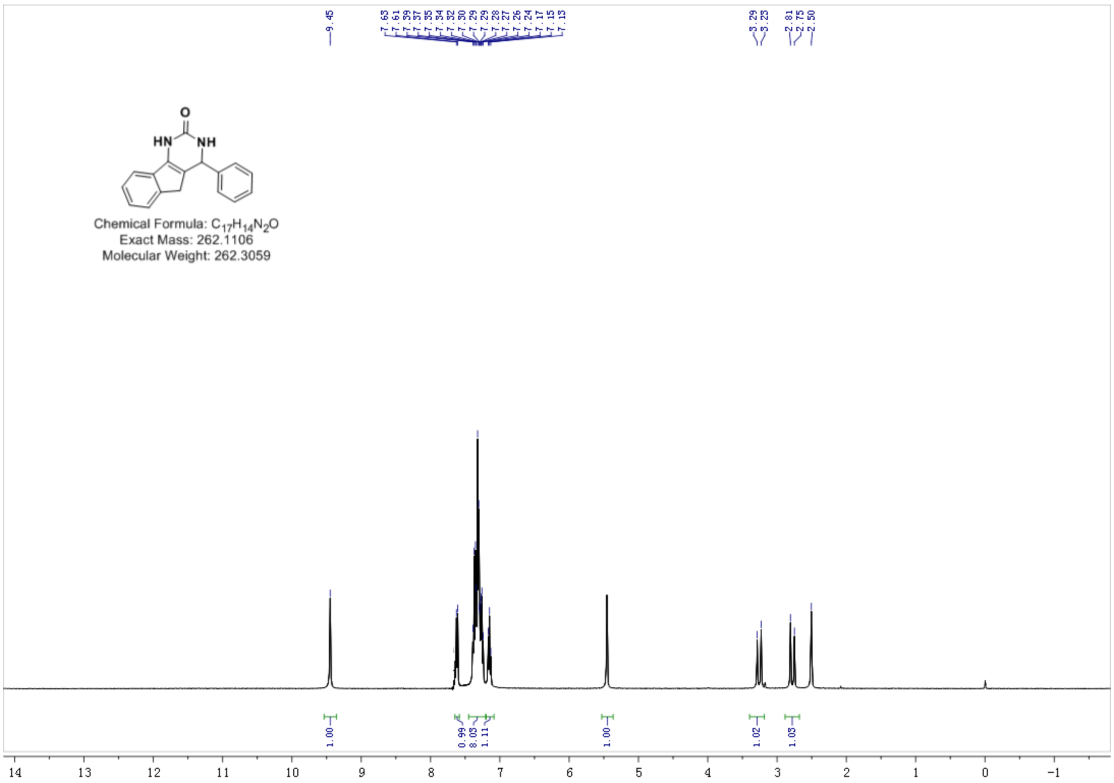

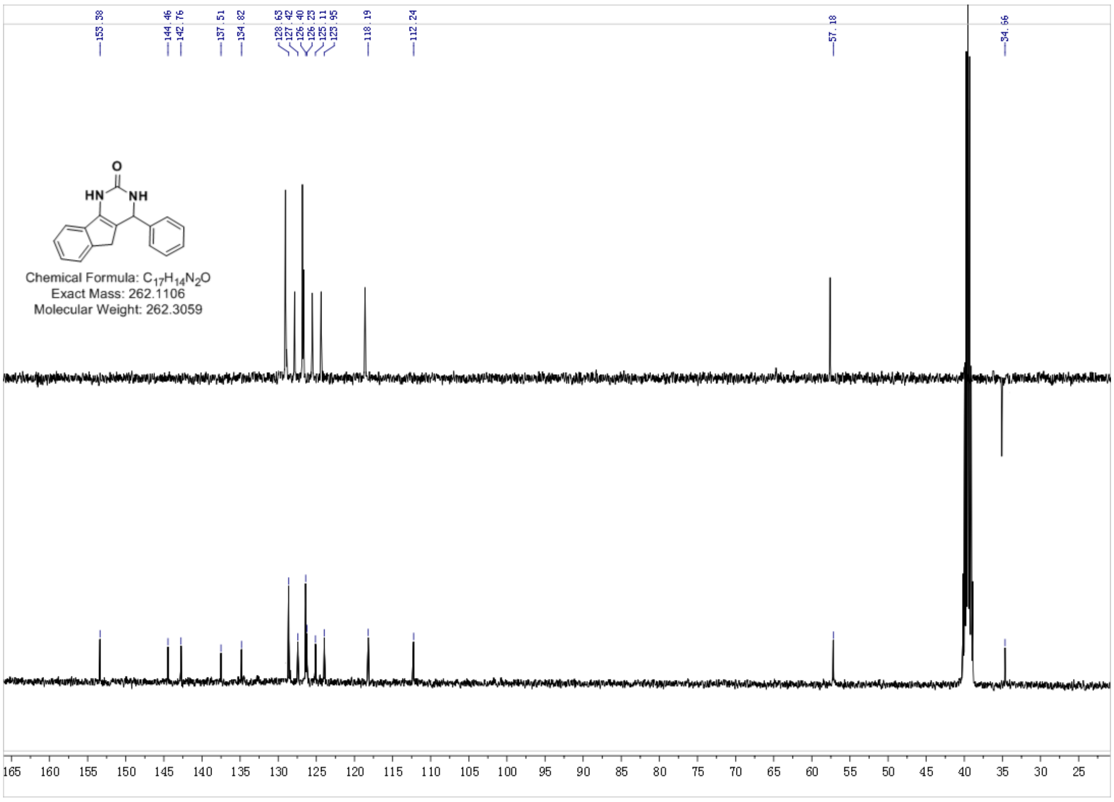

4-phenyl-3,4-dihydro-*1H*-chromeno[4,3-*d*]pyrimidin-2(*5H*)-one (6l)

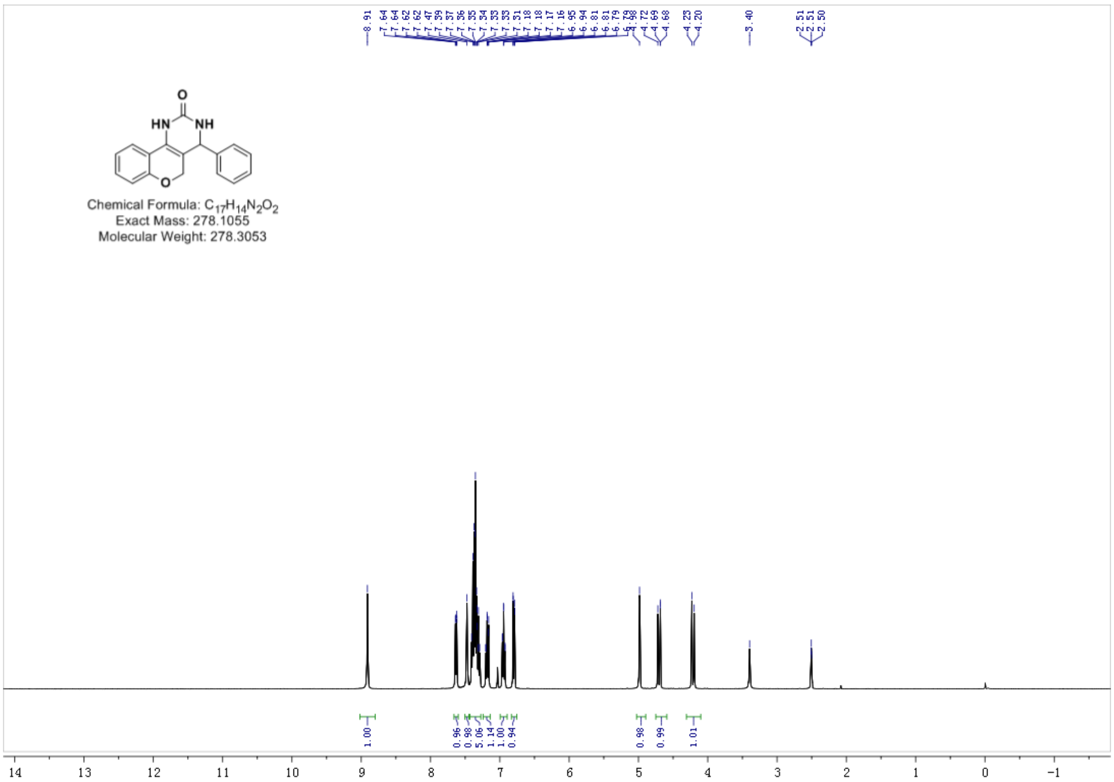

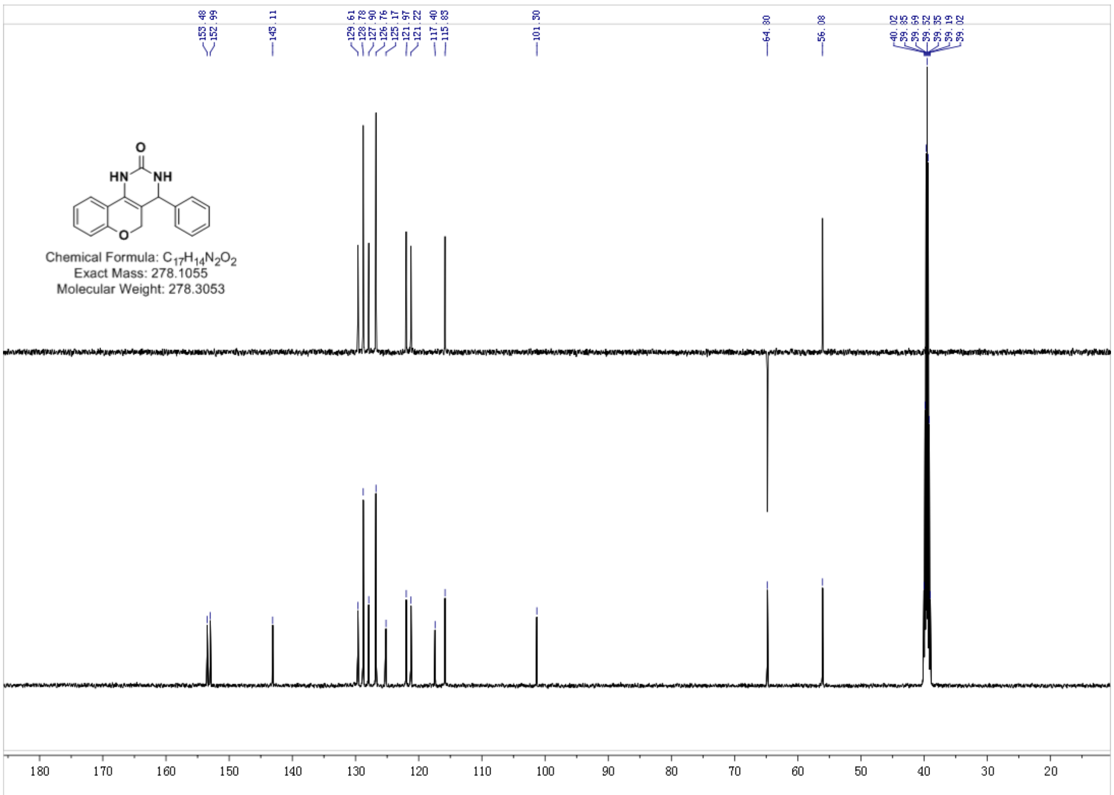

Supplement: Supplementary file 1 [file molecules-22-01503-s001.pdf]
